# A transition toward flightlessness in Mallards, Indian Runner Ducks, and their hybrid offspring

**DOI:** 10.1073/pnas.2534729123

**Published:** 2026-07-20

**Authors:** Ashley M. Heers, Willa Coultley, Sandy A. Gregorio

**Affiliations:** ^a^ Department of Biological Sciences, California State University, Los Angeles, CA 90032; ^b^ Department of Natural Sciences, Scripps and Pitzer Colleges, Claremont, CA 91711; ^c^ Department of Earth Sciences, University of Oregon, Eugene, OR 97403

**Keywords:** flightless birds, flightlessness, ontogeny, locomotion, heterochrony

## Abstract

Many studies have explored wing function in volant birds, and how flight is gained over ontogeny and evolution. However, flight is often secondarily reduced or lost, and how birds transition from wing- to leg-dependence is unknown. Most losses occurred long ago, so stages of wing reduction have never been functionally evaluated or even observed. Here, we assess the anatomical changes and locomotor consequences of a transition toward flightlessness in a unique group: flying Mallards (*Anas platyrhynchos*), flightless Indian Runner Ducks (Mallard-derivative), and their experimentally produced hybrid offspring. Based on wild birds, we hypothesized that shifts in development would lead to wing reductions in Hybrids and especially Runners, with compensatory increases in leg size and performance. Our results show that Runners indeed share many features with wild flightless birds, and that Hybrids are intermediate. However, the assembly of these features is surprising. Wings become relatively smaller due to increases in body size rather than reductions in wing area or muscle, which are conserved. Legs, in contrast, are labile: Runners invest proportionally less in their leg muscles as juveniles but more as adults than Mallards. This contrast between wing conservation and body and leg peramorphosis suggests that for at least some flight losses, bodies and legs change before wings, which get proportionally smaller before being reduced. Given that juveniles with incipient flight and adults with reduced flight both engaged their wings and legs cooperatively, wing-leg coordination may facilitate shifts along a flightless-to-flying spectrum, exemplifying how specialized structures can be gained or lost.

## Significance

Most birds are specialized for flight. Yet flight is often reduced or lost, and how this occurs is unknown because most losses occurred long ago, so stages of flight loss have never been observed. Here, we experimentally document developmental mechanisms behind a transition toward flightlessness in a unique group of ducks. Although flightless birds often have small, seemingly “useless” wings, our results suggest that small wings may initially result from increases in body size rather than reductions in wing size, and that birds incapable of sustained flight may still engage their wings and legs cooperatively. Such wing-leg coordination is also important to developing birds and shows how animals can use transitional behaviors as they gain or lose specialized structures.

Birds are well known for their flight. Flight is the most power-demanding mode of locomotion (1), and in many ways, the avian body plan revolves around these demands. For example, volant birds typically have large wings, hypertrophied pectoral muscles, and a robust skeleton (figure 1 in ref. 2), which collectively help meet flight’s requirements. These unique features result from one of the most dramatic transformations in vertebrate history, and many studies have examined how flight is gained during ontogeny and evolution (*SI Appendix*, *Text S1*), and how wings subsequently function in volant birds. However, flight is also commonly reduced or lost, and how wings function during this reverse process is unknown.

Flightlessness has probably evolved at least 150 times—possibly thousands of times, in over half of the avian orders (3–5). Familiar examples of secondarily flightless birds include ratites (ostriches and kin) and penguins (Sphenisciformes), along with more than 40 other extant species and several extinct groups (*SI Appendix*, Table S2 in ref. 6). There are also many cases of seasonal flightlessness, where birds temporarily lose flight capacity during feather molt or egg laying (*SI Appendix*, Table S1 in ref. 6). “Flightless” vs. “flight-capable” dichotomies are further blurred by examples of volant birds with reduced flight ability. For instance, flight capacity declines during ontogeny in some ground birds (Galliformes) and coots (Rallidae) (7–9), and many tropical species fly only short distances (10, 11). Losses and reductions in flight capacity are thus far more common and widespread than might be expected, given our instinctive association between birds and flight.

To make sense of this counterintuitive phenomenon, many studies have examined the habitats and morphological features associated with flight loss. Findings suggest that flight loss often requires 1) low predation rates, so flight is not needed for escape, and 2) a mild climate that does not require aerial migration to avoid seasonal hardships (4, 6). Indeed, the flight apparatus appears to be metabolically costly and may be reduced in birds that can transition to hind limb dependence when appropriate conditions are encountered (12–16). These transitions typically involve reductions in the flight apparatus (feathers, bones, and/or muscles), often resulting in an “underdeveloped,” juvenile-like anatomy (paedomorphosis), accompanied by increases in leg and body size (“overdevelopment” or peramorphosis) (Figure 5 in ref. 6). Because of such commonalities, both habitat and heterochrony, or shifts in developmental rate and/or timing, are well-recognized correlates of flight loss.

However, our understanding of how birds transition toward hind limb dependence as they lose flight capacity is hamstrung by a critical knowledge gap. Although many studies have examined wing function in volant birds, descriptions of wing function in semi-flightless or flightless birds are almost entirely anecdotal (6) (but see ref. 17), and stages of flight loss have never been observed. Consequently, shifts in morphology and locomotor performance have never been experimentally linked or even documented during transitions toward flightlessness. In some ways, this is not surprising. Flightless birds are often secretive and remote, and many lost flight so long ago that reconstructing their transition to flightlessness is difficult. Presumably, the flight apparatus was gradually reduced over evolutionary time. But what developmental changes lead to wing reduction, when wings have been under selection for flight? Young birds use their incipient wings to improve leg performance (*SI Appendix*, *Text S1*)—do wings similarly contribute to locomotion as wing size is reduced, or are they “useless” and even detrimental, as often supposed (6)? Do larger hind limbs compensate for reductions in forelimbs? These unanswered questions are essential for understanding the avian body plan and evolutionary transitions in flight capability. For, despite our instinctive bias toward flight, birds clearly shift along a flightless-to-flight-capable spectrum (6). Yet, we do not really know how organisms lose specialized structures that have become so central to their lives. In birds, this is because the stages of wing reduction and accompanying shifts to leg dependence have never been observed or functionally quantified.

Here, we experimentally created a transition toward flightlessness, tracking anatomical and locomotor development across a unique spectrum of ducks: flight-capable Mallards (*Anas platyrhynchos*), flightless Indian Runner Ducks (a domesticated Mallard derivative), and, after breeding, their hybrid offspring. While many domesticated birds are bred for meat production and live in highly artificial conditions, Indian Runner Ducks do not. Traditional duck herding practices and both early and modern records (details and citations in *SI Appendix*, *Text S2*) indicate that Runner Ducks:

- likely originated in the Malay Archipelago, possibly more than 1,000 y ago—but much more recently than wild flightless birds
- have been prized for egg production: eggs were eaten or incubated, and juveniles were reared by humans and often walked long distances to market
- have been valued for pest control—traditional practices involve leading ducks out to fields or rice paddies, where they forage over long distances
- are highly active, terrestrial foragers that rarely use their wings and walk rather than waddle (without marked side-to-side swaying to shift body mass over the supporting foot), using an upright, penguin-like posture
- readily hybridize with other Mallard-derivative ducks, which presumably could yield “semi-flight-capable” hybrid offspring

Runner Ducks thus represent a recently flightless, highly active, outdoor terrestrial bird. We leveraged these unique characteristics to experimentally document a reduction in flight capacity by breeding flying Mallards with flightless Runners to produce hybrid offspring (herein “Hybrids”). This approach yielded a wide spectrum of flying to flightless birds. Our intent here is to compare anatomical and locomotor ontogeny across these three groups to 1) quantify how wing and leg performance change as wings are reduced, and 2) illuminate developmental mechanisms underlying wing reduction. We hypothesized that shifts in developmental rate and/or timing (heterochrony) in Runners ± Hybrids would result in wing reductions (e.g., paedomorphosis) accompanied by compensatory increases in leg investment (peramorphosis) and performance, as observed in many wild birds (6). To test this, we measured wing and leg morphology (wing, feather, and foot areas; muscle masses; bone lengths) and performance (running, flying, and swimming velocity; aerodynamic force production) in all three groups, expecting Mallards to be the most flight-capable and Runners the least.

## Results

Mallards, Runners, and Hybrids visibly differed in both anatomy and behavior. Runner Ducks had long, slim bodies, straight necks, and an upright posture compared to Mallards (*SI Appendix*, Fig. S1). Hybrids were variable but generally intermediate in posture and size. During experiments, birds were kept in identical indoor enclosures, and differences in activity levels or behavior were not observed. However, differences emerged after experiments ended, when birds were transferred to outdoor enclosures with pools and elevated surfaces. Mallards and some Hybrids were infatuated with water and always swimming, whereas Runners were the most motivated by food. Mallards and sometimes Hybrids flew up to and down from elevated surfaces; Runners usually walked, but did use their wings to jump for food or swim, and occasionally to elude capture.

Beyond these general differences in morphology and behavior, we found significant differences in body mass, wing and leg anatomy, and wing and leg performance. All group differences described below are statistically significant at *P* < 0.05 (*SI Appendix*, Table S1) unless specified as “likely.” Although these findings overall were consistent with previous work on Mallards (18) and generally aligned with our expectations, their developmental underpinnings were, in many cases, surprising.

### Body Size: Hybrids and Especially Runners Get Big by Growing Faster, Longer (*SI Appendix*, Tables S1 and S2).

**Fig. 1.**
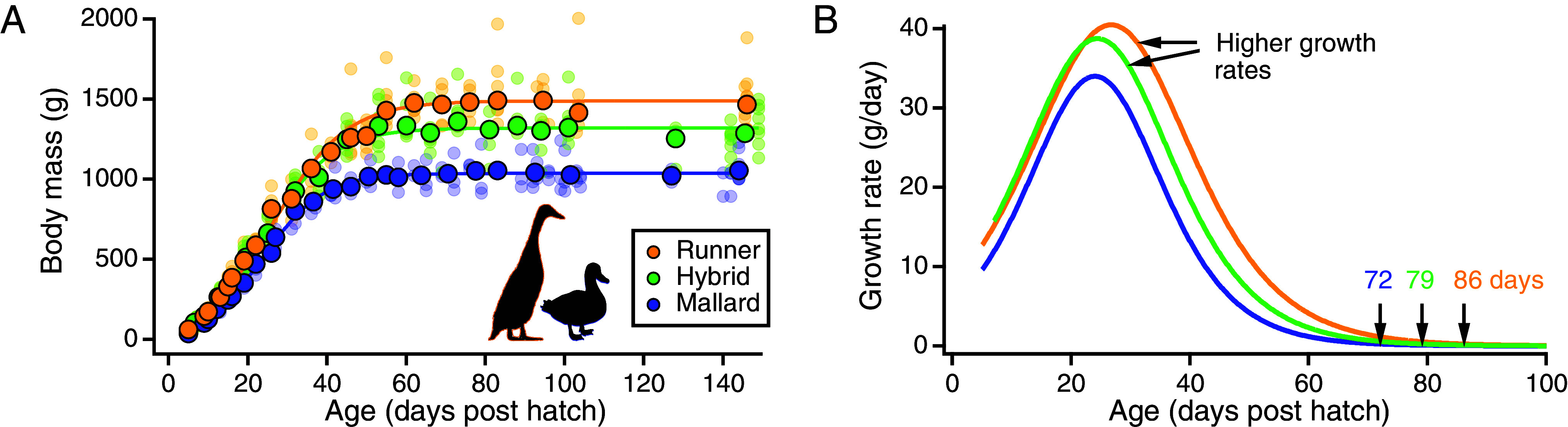
Body Size: Hybrids and especially Runners get big by growing faster, longer. (*A*) Body mass and (*B*) growth rates. Light circles = individual data points; dark circles = averages. Growth rates in (*B*) are the derivatives of the logistic growth curves in (*A*). Silhouettes in public domain. Statistical outcomes and mean values in *SI Appendix*, Tables S1 and S2.

Wild flightless birds often have larger bodies than their predecessors (6), and our ducks showed a similar pattern. Runners reached higher body masses than Mallards, and Hybrids were intermediate, due to faster growth rates and extended growth periods (Fig. 1). These differences in body mass ultimately impacted relative limb investment (e.g., wing or foot area, muscle mass) and performance.

### Wing Anatomy: The Flight Apparatus Is Proportionally Smaller, but not Reduced, in Runners ± Hybrids.

Given that wild flightless birds also commonly have a proportionally small and paedomorphic flight apparatus (6), we expected Runners to have one as well. Relative investment in the flight apparatus did differ between Runners and Mallards, and to some extent between Hybrids and Mallards, largely as predicted. However, growth of the flight apparatus was surprisingly conserved, and differences were instead due to differences in body size.

#### Wing area: wings are proportionally smaller, but not reduced, in Runners (*SI Appendix*, Tables S1 and S3).

**Fig. 2.**
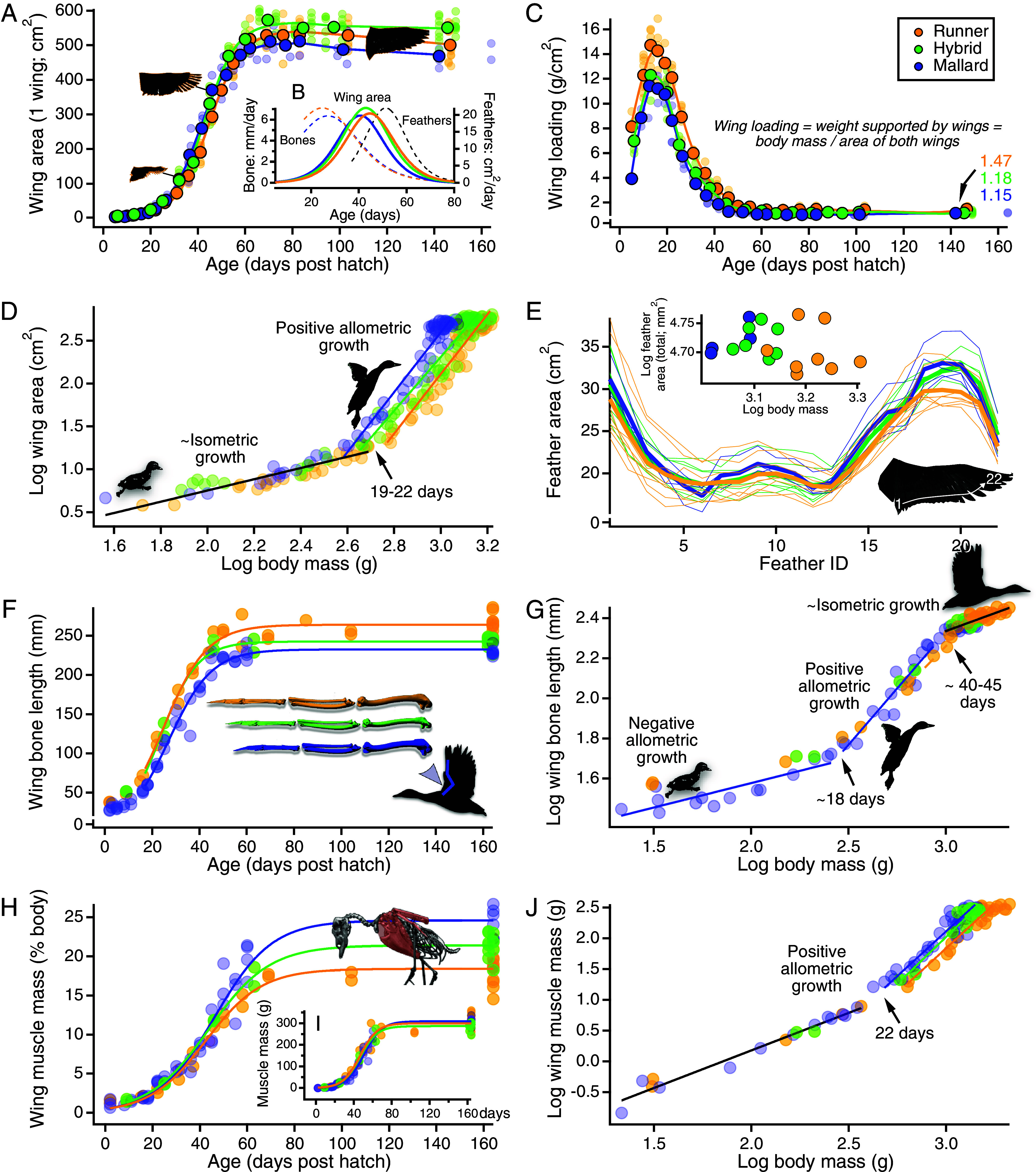

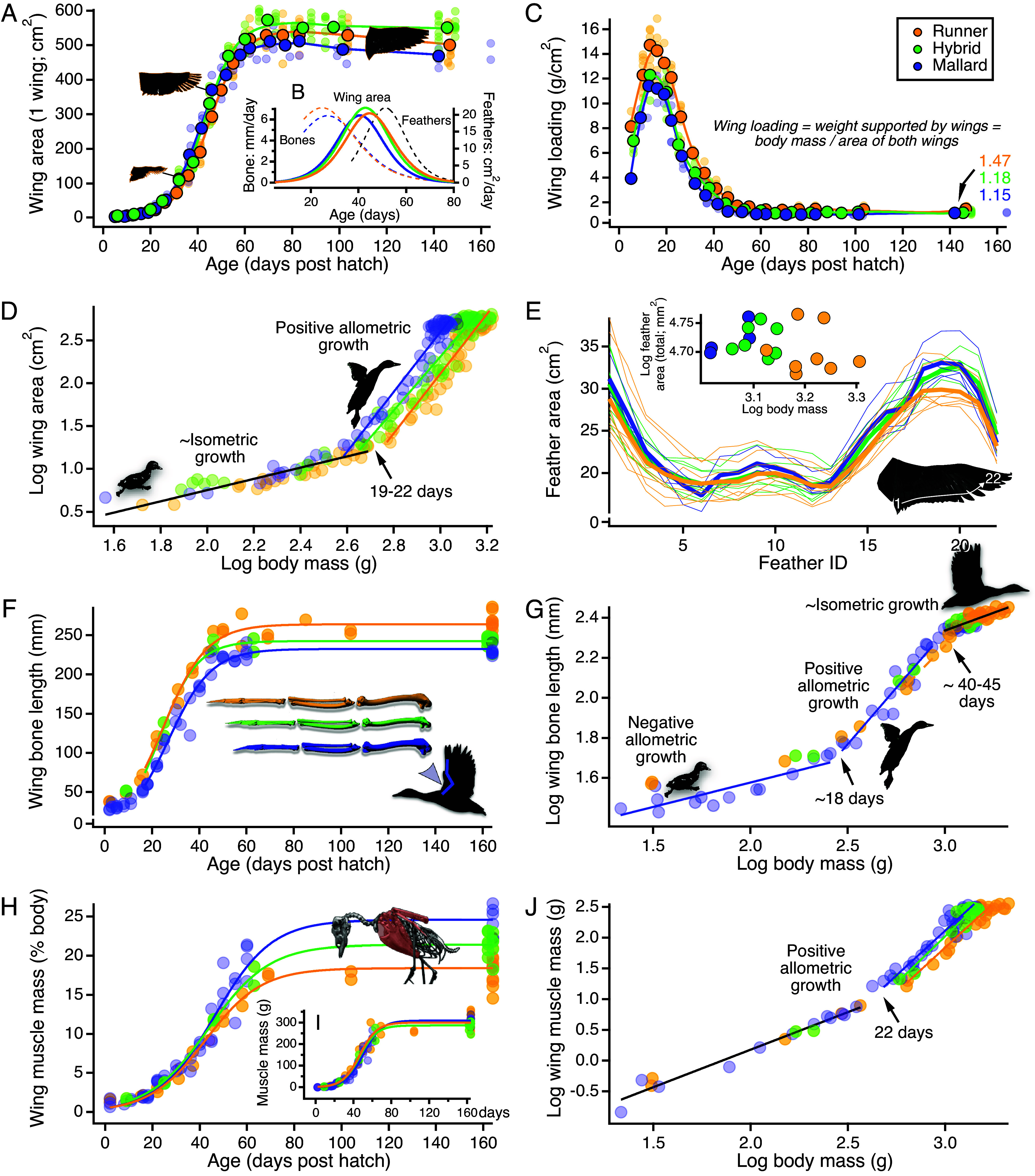
Wing Anatomy: wing development is delayed (18 to 22 d) and the flight apparatus is proportionally smaller, but not reduced, in Runners ± Hybrids. (*A*) Wing area and (*B*) growth rates are not reduced in Hybrids or Runners, but area (*C*) is proportionally smaller in Runners, which (*D*) begin growing their wings at approximately the same age but at larger body sizes. (*E*) Wing feather areas (primaries–secondaries) and scaling (*Inset*), vs. (*F*) wing bone length (humerus + ulna + carpometacarpus) and (*G*) scaling: feather area is approximately conserved, except for reduced distal primaries in Runners, whereas bone length increases with adult body size and explains the larger wings of Hybrids and maintained wings of Runners. Though we did not have enough Hybrids and Runners to analyze early bone growth, their positive allometric growth may be initiated at larger body sizes (shifted right; dashed line), as with wing area (*D*). (*H*) Wing muscles (all muscles acting on the shoulder, elbow, or wrist; Left + Right sides) are proportionally smaller in Hybrids and especially Runners, but (*I*) not reduced; (*J*) all groups begin rapidly growing muscles at approximately the same age, when Hybrids and Runners are already at larger body sizes. Light circles = individual data points; dark circles = averages; lines = logistic growth curves (*A*, *F*, *H*, and *I*), derivatives of logistic growth curves (*B*; Hybrid bones not shown due to small sample size), linear regressions (*D*, *G*, and *J*), smoothing splines (*C*), or bolded averages (*E*). Mallard bones and muscles supplemented from (18). Mounted skeleton, and silhouette in (*F*), in public domain. Statistical outcomes, scaling slopes, and mean values in *SI Appendix*, Tables S1 and S3–S5.

As expected for waterfowl (19), Runners, Mallards, and Hybrids had delayed wing growth and use. However, contrary to our initial expectations for a flightless bird, Runners did not have reduced wing areas compared to Mallards (Fig. 2*A*), and Hybrids surprisingly grew larger wings by incorporating both Mallard- and Runner-like growth rates (Fig. 2*B*). Though Runners did have the proportionally smallest wings (highest wing loading) (Fig. 2*C*), this resulted from relatively conserved wing growth (Fig. 2 *A* and *B*) coupled with greater body growth (Fig. 1): Runners initiated similar rates of rapid wing growth (positive allometry) at approximately the same age as Mallards and Hybrids (19 to 22 d), but at a larger body size (Fig. 2*D*). The proportionally smaller wings of Runners are therefore due to increases in body size rather than reductions in wing size. Hybrids, in contrast, resembled “big Mallards” (similar wing loadings), due to intermediate rates of body growth and rapid and prolonged wing growth.

#### Bone versus feather growth: bones track body size and precede feathers, which are relatively conserved (*SI Appendix*, Tables S1, S3, and S4).

Wing area depends on bone length and feather area, which are often both reduced in flightless birds (6), but were not reduced in Hybrids and were only partially reduced in Runners. Like other birds with late wing development (20), increases in wing area were initially due to bone growth and later to feather growth (Fig. 2*B* and *SI Appendix*, Fig. S2). Hybrids grew their larger wings by having Mallard-like feathers anchored to slightly longer forelimbs, whereas Runners had smaller primary feathers but maintained large wings by having the longest forelimbs (Fig. 2 *E* and *F* and *SI Appendix*, Fig. S3). These longer forelimbs resulted from higher growth rates (Fig. 2*B*) and isometric scaling (Fig. 2*G*): following periods of negative and then strongly positive allometry, bones grew isometrically such that the largest birds had the longest forelimbs (Runners > Hybrids > Mallards). This contrasts with feather area, which did not scale with adult body mass (Fig. 2 *E*, *Inset*). Thus, bone growth occurred earlier and scaled with body mass, whereas feather growth was approximately conserved except for some distal primaries, which were reduced in Runners. This suggests that although Runners and Hybrids retain large wings, their wing feathers overlap less with each other, which might compromise performance.

#### Muscle mass: wing muscles are conserved, but proportionally smallest in Runners (*SI Appendix*, Tables S1 and S5).

As expected for a flightless bird, Runners had the proportionally smallest wing muscles, Mallards had the largest, and Hybrids were overlapping and intermediate (Fig. 2*H*). However, as with wing area, neither Runners nor Hybrids had reduced muscle growth (Fig. 2*I*). Instead, they initiated similar rates of rapid muscle growth (positive allometry) at the same age as Mallards (~ 22 d) but again at larger body sizes (Fig. 2*J*), resulting in proportionally smaller wing muscles.

These patterns collectively show that, like wild flightless birds, Hybrids and especially Runners have a proportionally smaller flight apparatus than their flight-capable relative. However, this shift was due to increases in body size (peramorphosis) rather than reductions in the flight apparatus (paedomorphosis). Body-driven differences in relative wing investment nevertheless manifested as differences in wing performance.

### Wing Performance Mirrors Relative Wing Investment: Mallards > Hybrids > Runners.

Wing performance was measured during controlled flapping descent (descending flight; can be quantified early in ontogeny) and vertical takeoff (ascending flight; pushes locomotor limits). Performance was quantified as 1) vertical aerodynamic force production (in multiples of body weight; standardized by number of wingbeats for vertical takeoff–*SI Appendix*, Fig. S4), ± 2) vertical flight velocity. Unsurprisingly, Mallards performed the best. However, Hybrids and Runners did better than anticipated.

#### Controlled flapping descent: Mallards & Hybrids > Runners, with Hybrids and Runners performing better than expected—presumably due to conserved wing size (*SI Appendix*, Table S1).

**Fig. 3.**
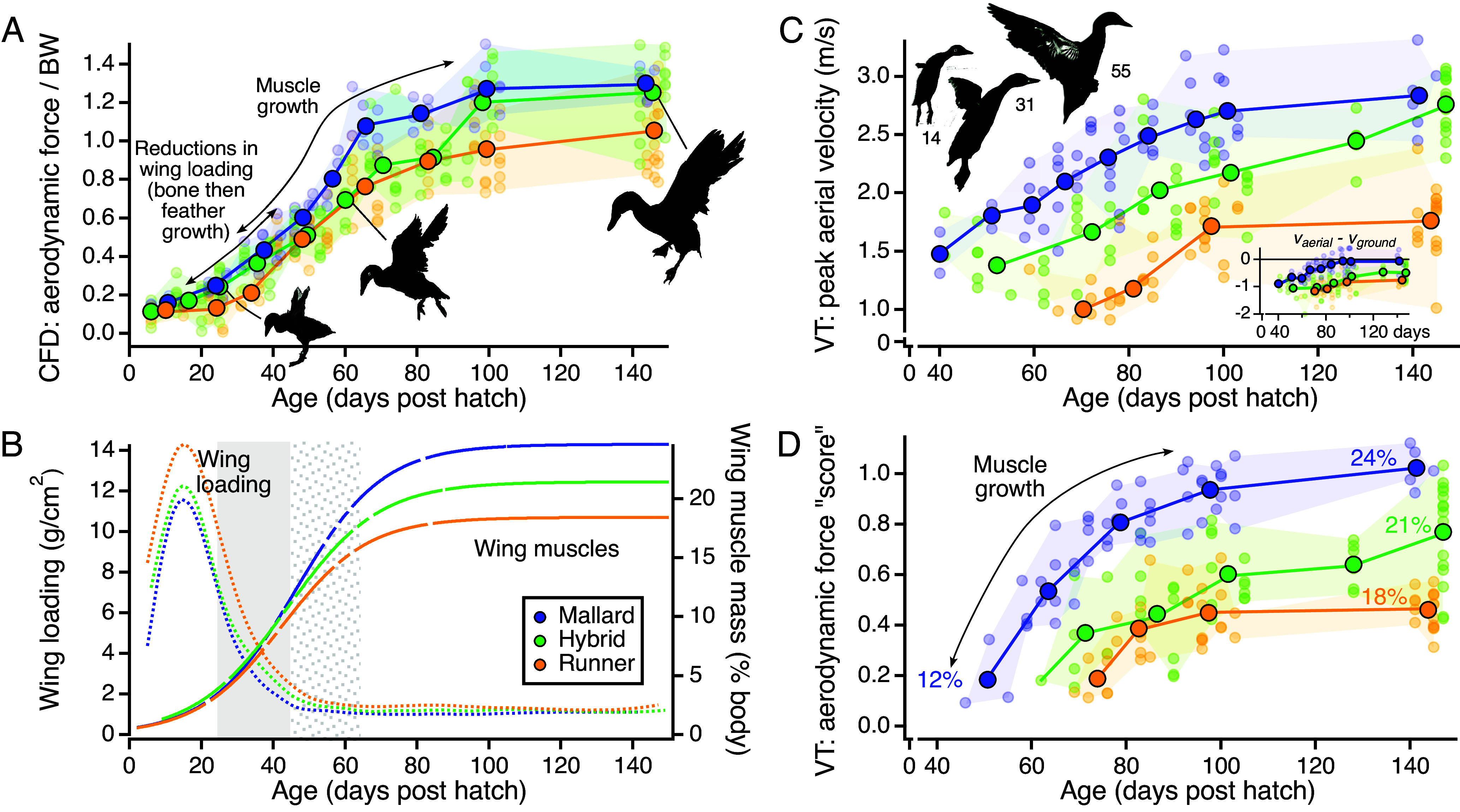
Wing performance mirrors relative wing investment: Mallards > Hybrids > Runners. (*A*) Ontogenetic improvements in wing performance during controlled flapping descent (CFD) track (*B*) reductions in wing loading and then increases in muscle mass. (*C* and *D*) Vertical takeoff (VT) is more difficult and begins later, with improvements tracking muscle development. In Hybrids, VT velocities are somewhat inflated by their high leg performance (*C*, *Inset*: aerial–ground velocity), and VT scores continue to improve very late in ontogeny, potentially due to increasing confidence: whereas Mallards were always “confident” in flight and Runners were always reluctant, Hybrids seemed to gain confidence with age and were increasingly willing to attempt difficult flights. Light circles = individual data points; dark circles = averages; lines in (*B*) from Fig. 2 and solid or hashed bars from Fig. 5. BW = body weight; % in (*D*) = average wing muscle mass percents from Fig. 2; VT score = (aerodynamic force/BW) (# wingbeats + 3)/6—this penalized birds slightly for completing fewer than three wingbeats but maintained the general ontogenetic patterns; *SI Appendix*, Fig. S4. Statistical outcomes in *SI Appendix*, Table S1.

Controlled flapping descent (CFD; Movies S1 and S2) allows birds to slow an aerial descent (21) and is particularly relevant to ducklings, which may hatch in elevated nests (22). Our ducklings were indeed quite willing to leap from platforms and typically flapped as they descended, though their small wings probably did not generate much lift. However, ducklings also spread their webbed feet, and this, combined with their downy feathers and flapping wings, likely increased drag production to help slow descents. Aerodynamic performance improved throughout ontogeny in all three groups (Fig. 3*A*). These improvements initially tracked reductions in wing loading, and later the growth of wing muscles (Fig. 3*B*).

Hybrid performance was variable and overlapped with that of Mallards more than expected. This is probably because Hybrids had large wings, and slowing an aerial descent presumably requires less muscle power than other flight behaviors. Runners, on average, had lower wing performance than Hybrids and Mallards, but also better than expected—again, probably due to their conserved wing size.

#### Vertical takeoff: Mallards > Hybrids > Runners—mainly due to differences in wing muscle mass, though Runners again exceeded expectations (*SI Appendix*, Table S1).

Vertical takeoff (VT) is more challenging than descending flight (CFD). Because wing development is delayed in ducks, this behavior was thus initially more of a jump (Movies S3 and S4). Wings began to contribute measurably at approximately 40 d in Mallards and 70 d in Runners, with Hybrids falling in between (Fig. 3 *C* and *D*). Wing loading was already low at this point and did not decline much further (Fig. 3*B*). Wing muscles were just exceeding approximately 10% body mass, and improvements in VT were associated with continued muscle growth.

Like descending flight (CFD), Mallards had the highest wing performance, Runners had the lowest, and Hybrids were variable and intermediate. However, these differences were more substantial than those observed during CFD, presumably because VT requires greater muscle power and pushes locomotor limits. Similarly, differences in VT force production were more pronounced than differences in VT velocity, probably because force was measured over multiple wingbeats and therefore reflects both peak performance and endurance. In all cases, though, Runners performed better than expected and clearly used their wings to enhance their jumps.

Thus, Mallards have the best wing performance, but Runners and Hybrids performed better than predicted and were able to get off the ground and slow aerial descents.

### Leg Anatomy: Hybrids and Especially Runners Invest More in their Hind Limbs.

Due to delayed wing development, ducks are dependent on their hind limbs as juveniles, and legs showed earlier and steadier growth than wings. Because wild flightless birds often have large (peramorphic) legs (6) that potentially compensate for their reduced wings, we expected Runners to have the highest leg investment and Mallards the lowest. Although this prediction was met overall, leg development differed across groups in unexpected ways.

#### Foot area: Runners have large feet, and Hybrids have proportionally small feet (*SI Appendix*, Tables S1, S2, and S6).

**Fig. 4.**
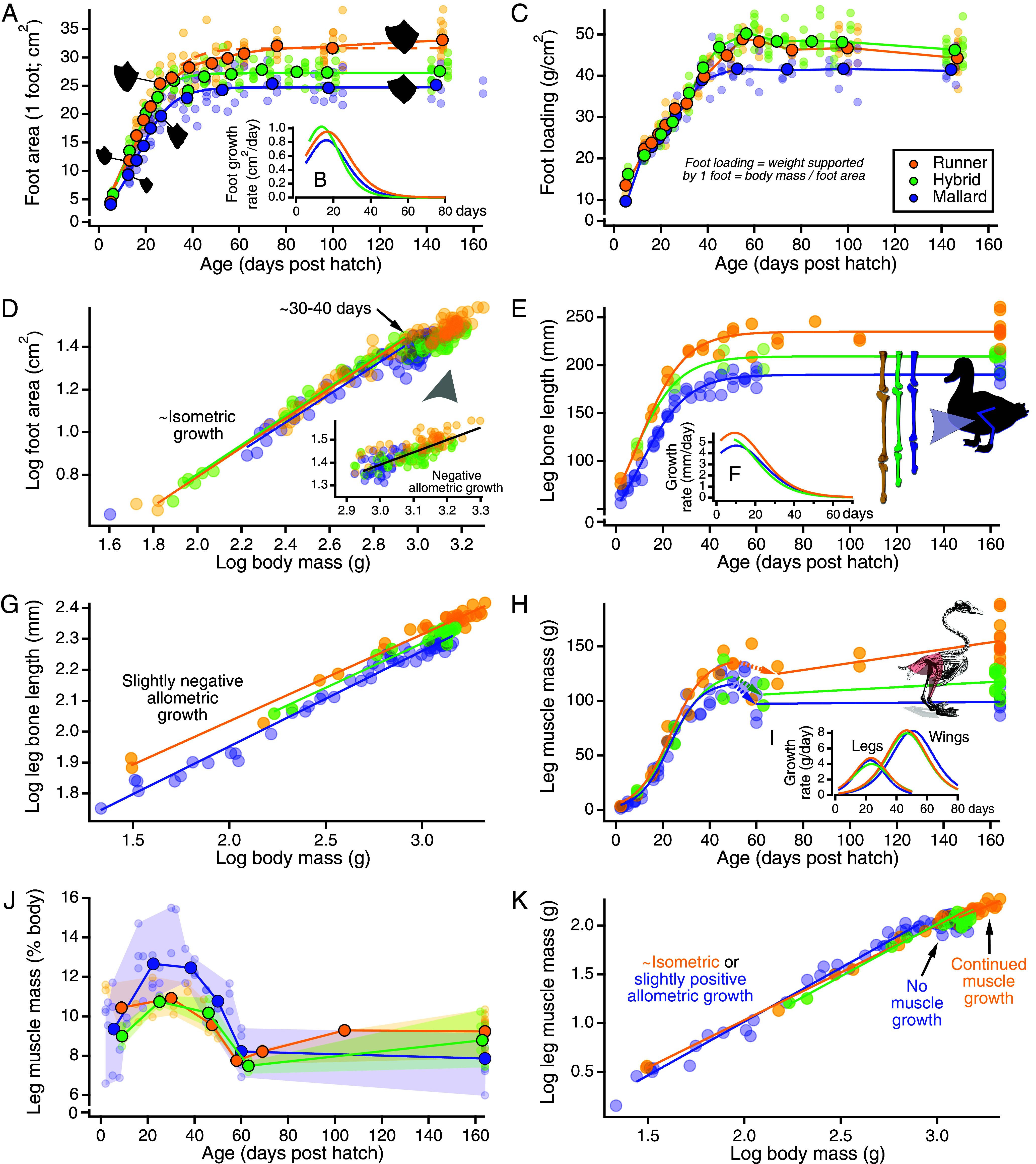
Leg anatomy: legs develop early, and Hybrids and especially Runners invest more in their hind limbs. (*A*) Runners have large foot areas due to (*B*) faster and prolonged growth, whereas Hybrid adults have feet that are (*C*) proportionally small for their body size, due to (*B* and *D*) a shorter period of foot growth relative to body growth (large, Runner-like feet at small body sizes, but smaller feet than Runners of the same size as adults). (*E*) Hybrids and especially Runners have longer leg bones (femur + tibiotarsus + tarsometatarsus), due to (*F*) faster growth, at (*G*) all body sizes. (*H*) Leg muscles (all muscles acting on the hip, knee, or ankle; Left + Right sides) grow rapidly, then get resorbed when (*I*) wing growth is high. (*J*) This decline is most extreme in Mallards, which have proportionally larger leg muscles as juveniles but smaller leg muscles as adults. Hybrids and especially Runners regain leg muscle after wing growth slows, whereas in older Mallards, (*K*) leg muscle does not increase with body mass. Light circles = individual data points; dark circles = averages; lines = logistic growth curves (*A*, *E*, and *H*), derivatives of logistic growth curves (*B*, *F*, and *I*), linear regressions (*D*, *G*, and *K*), or smoothing splines (solid orange curve in *A*). Mallard bones and muscles supplemented from (18). Duck skeleton in public domain. Statistical outcomes, scaling slopes, and mean values in *SI Appendix*, Tables S1 and S4–S6.

Consistent with peramorphosis, Runners grew the largest feet (Fig. 4*A*), by having higher growth rates and longer growth periods (Fig. 4*B*). Hybrid feet, though slightly larger than Mallards, were actually proportionally smaller for their body size (higher foot loading) (Fig. 4*C*). This seems to stem from Hybrids having a shorter period of foot growth relative to body growth (Fig. 4*B*): Hybrids had large, Runner-like feet at small body masses, but stopped foot growth earlier and tended to have smaller feet than Runners of the same mass as adults (Fig. 4 *A* and *D*). Runners, in contrast, had large feet at all body sizes. However, since foot size scaled with slight negative allometry in older ducks, foot loading increased with body size and, in older Runners, overlapped with Hybrids and Mallards. Thus, Runners had large feet but not proportionally so, whereas older Hybrids had proportionally small feet.

#### Bone length: Hybrids and especially Runners have longer legs (*SI Appendix*, Tables S1 and S4).

Also consistent with expectations, Runners had longer legs than Mallards as adults (Fig. 4*E* and *SI Appendix*, Fig. S5), mainly due to higher growth rates (Fig. 4*F*). Hybrids were intermediate. These differences held across body sizes (Fig. 4*G*), although young Runners and Hybrids had similar leg lengths at a given age, and adults were somewhat less leggy than juveniles due to slightly negative allometric growth. Hybrids and Runners had longer forelimbs than Mallards as well, but to a lesser extent, reiterating their high leg investment.

#### Muscle mass: leg muscles grow early but are resorbed—especially in Mallards, then regrown in Hybrids and Runners (*SI Appendix*, Tables S1 and S5).

As might be expected for a precocial bird, leg muscles grew rapidly early in ontogeny (Fig. 4*H*). However, at approximately 50 to 60 d, leg muscle mass declined. Presumably, leg muscle was resorbed during rapid wing muscle growth (Fig. 4*I*)—similar reallocations occur in wild birds that simultaneously molt and then regrow all of their wing feathers (*SI Appendix*, Table S1 in ref. 6). In addition, patterns differed across Mallards, Hybrids, and Runners. Mallards likely had the proportionally largest leg muscles as juveniles, followed by the steepest decline (Fig. 4*J*). After this decline, Mallard leg muscles remained steadily small. In contrast, Hybrids and especially Runners appeared to regain leg muscle. Indeed, in young Mallards, leg muscles scaled more steeply than Hybrids and Runners, whereas in older Mallards, body mass increased, but leg muscle did not (Fig. 4*K*).

These differences in leg investment coincide with other differences in resource allocation. Runners and likely Hybrids had proportionally smaller leg muscles as juveniles but invested more in overall body growth. Following resorption, Hybrids and Runners regained leg muscle, after wing growth slowed. Mallards did not, but they are more wing-dependent, and keeping leg “baggage” light is likely advantageous.

Taken together, these patterns show that legs are labile, resulting in both expected and unexpected differences that were, in turn, associated with differences in leg performance.

### Leg Performance Tracks Leg Development, Increasing Then Decreasing—Especially in Mallards (*SI Appendix*, Table S1).

**Fig. 5.**
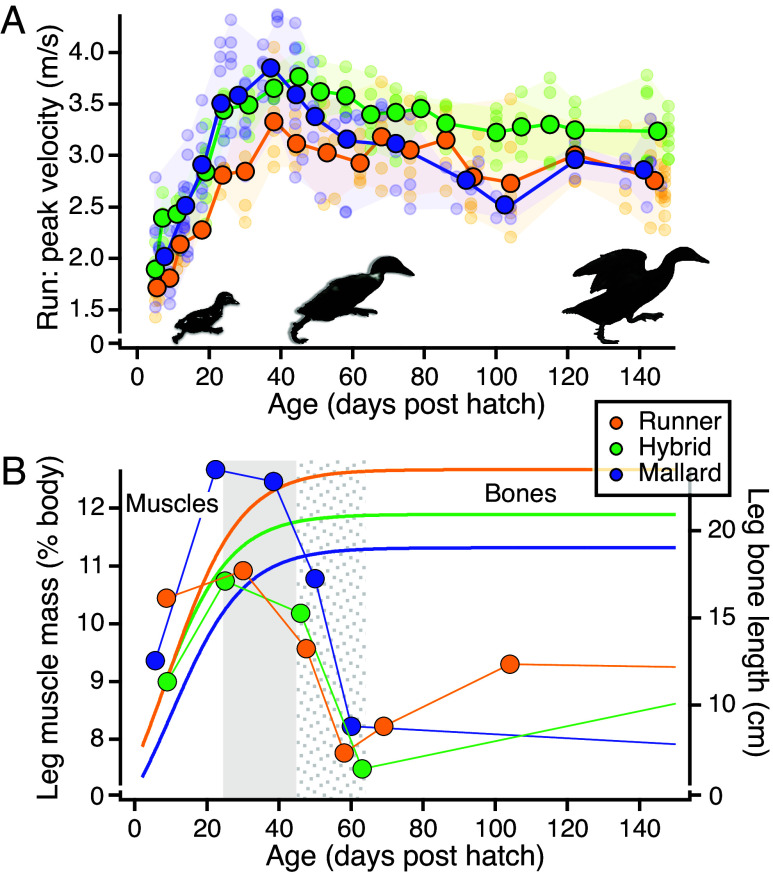
Leg performance tracks leg development, increasing then decreasing—especially in Mallards. (*A*) Running performance tracks (*B*) leg investment and peaks early in ontogeny, then declines. This decline is the most extreme in Mallards. Light circles = individual data points (similar values in ref. 23), dark circles = averages; lines in (*B*) from Fig. 4; solid gray bar in (*B*) and Fig. 3 = period of peak leg performance, hashed bar = declining leg performance. Mallard performance supplemented from (18). Statistical outcomes in *SI Appendix*, Table S1.

Leg performance was measured during running (Movie S5; *SI Appendix*, Text S3), which is important to ducklings and potentially to flightless birds. Running velocity tracked leg development, increasing rapidly after hatching and peaking around 25 to 45 d (Fig. 5*A*), when legs were nearly at full length and leg muscles were proportionally their largest (Fig. 5*B*). Performance then declined as legs became less muscular and wings became more muscular and capable (Fig. 3). This decline was the most extreme in Mallards, which had proportionally the most muscular legs and fastest running as juveniles, but the least muscular legs and slower running in adulthood.

As adults, Hybrids had the best running performance, and Runners were similar to Mallards. This was somewhat surprising, given that adult Runners had the longest legs and largest muscles. One possibility is that Runners are not very athletic due to domestication. However, we suspect that Runners’ slower-than-expected speeds may be related to their upright posture and large feet, which might improve locomotor efficiency but limit top running speeds (*Discussion*).

In short, Mallards performed the best as juveniles, but Hybrids ran faster than Mallards as adults.

### Cooperative Use of Wings and Legs.

Overall, results aligned with some, but not all, of our expectations. One of the first and most obvious surprises was the level to which Runner Ducks could use their wings. Accounts of wing function (or lack thereof) in poorly flying and “flightless” birds are rare and almost all anecdotal (6), and the implicit assumption seems to be that flightless birds do not use their wings during locomotion. Although wing performance was indeed lower in Hybrids and especially in Runners, both groups still utilized their wings. In addition, all of our ducks engaged their wings and legs cooperatively. Whether more wing-invested or leg-invested, this wing-leg coordination resulted in continuous ontogenetic improvements in whole-body performance, rather than delays (as with flight) or declines (as with running). This was observed during three behaviors:

#### Steaming: using legs like paddles and wings like oars.

**Fig. 6.**
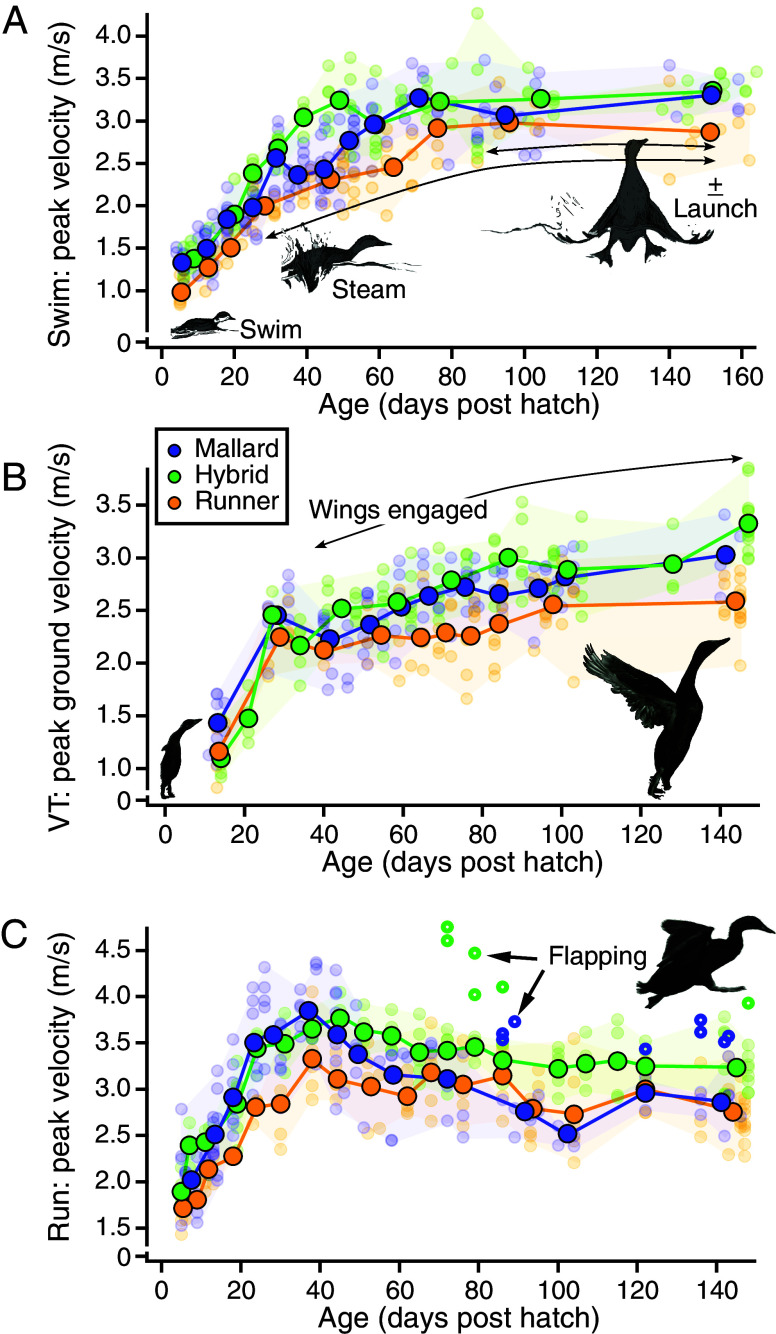
Coordinated use of wings and legs. When ducks engage their wings and legs together to (*A*) steam, (*B*) leap into the air, or (*C*) leap along the ground, performance continually improves rather than showing delays (as with flight) or declines (as with running). Engaging the wings while running likely improves both speed and balance. Light or open circles = individual data points; dark circles = averages. Illustrations in (*A*) by Bob Petty, modified from (6).

Steaming (Movie S6) is common in aquatic birds (7, 17, 24, 25). Ducklings initially swam using only their legs (Fig. 6*A* and *SI Appendix*, Fig. S6), but juveniles began using their wings to steam around 30 to 40 d, coincident with improvements in controlled flapping descent and the beginnings of vertical takeoff. By 90 d, some ducks also used their wings and legs to launch themselves out of water. Thus, wing contributions to swimming increased with age, and all three groups showed approximately continuous improvements.

#### Wing-assisted jumps transition into vertical takeoffs.

Birds also use their wings and legs cooperatively to jump and launch themselves into the air (26) (Fig. 6*B*). Initially, ducklings leapt without engaging their wings. Vigorous flapping began around 40 d, at first at the very end of the jump phase but progressively earlier as the wings developed. As with steaming, jumping or takeoff velocity improved approximately continuously throughout ontogeny.

#### Flap-running.

Older Hybrids and Mallards sometimes gave a burst of wing flapping while running. This resulted in 1 to 3 long leaps and an increase in velocity (Fig. 6*C*). Presumably, these birds would have flown instead had they been outdoors. However, juvenile Killdeer (*Charadrius vociferus*) appear to regularly engage in flapping runs, and similar behaviors have been proposed for some extinct theropods (27).

Collectively, these three behaviors reiterate previous work showing that coordinated use of wings and legs can mitigate potential tradeoffs between them (*SI Appendix*, Fig. S7), such that whole-body performance improves throughout ontogeny despite differences or developmental offsets in wing and leg investment (9). For example, running performance begins to decline at approximately 45 to 50 d in conjunction with developmental declines in leg investment (Fig. 5). However, wing investment is increasing at this point (Fig. 3*B*). Thus, when wings and legs are engaged cooperatively, wing contributions likely compensate for reductions in leg investment, allowing whole body performance to continue to improve. This is true even though Mallards, Hybrids, and Runners have different ratios of wing-to-leg investment, because wings and legs complement each other during such behaviors. Given that juveniles with incipient flight (*SI Appendix*, Text S1) and adults with reduced flight (Fig. 6) both engaged their wings and legs cooperatively, wing-leg coordination may facilitate gradual gains and losses of flight—by allowing wings to continuously contribute to locomotor performance as birds shift along a spectrum of wing-to-leg dependence.

## Discussion

Flight has been reduced or lost many times in birds (3, 4). While a multitude of studies have explored how flight is gained and how wings subsequently function in volant birds, the reverse is not true. How wings function as they are reduced is unknown, because a transition from wing- to leg-dependence has never been observed or functionally quantified. This partially stems from the difficulty of finding closely related wild birds that span a wide range of flight capabilities. Domesticated birds and hybridization can help address this challenge, though not without limitations. For example, artificial selection or captive breeding may produce phenotypes that are maladaptive in wild populations, and hybrids may exhibit maladaptive trait combinations and behaviors that would not be expected in an evolutionary intermediate (28, 29). Nevertheless, our Runners and Mallards shared many similarities with their wild counterparts, and although our Hybrids displayed some unexpected character combinations, they 1) maintained high overall locomotor performance (e.g., fastest running), 2) shared several features with wild semi-flightless birds (e.g., intermediate wing and leg morphologies), and 3) displayed a range of phenotypes that will facilitate detailed examination of form–function relationships across a spectrum of wing and leg anatomy. In short, Mallards, Indian Runner Ducks, and their intermediate hybrid offspring collectively span a wide range of flight capacities and provide insight into secondary reductions in flight capacity by illuminating possible developmental mechanisms of wing reduction and some conditions and behaviors that may facilitate this process.

### Developmental Mechanisms of Flight Loss.

Flightless birds are well known for having a small flight apparatus (feathers, bones, and/or muscles) (6). How does the process of wing reduction begin, when wings have been under such strong selection for flight? Given that many flightless birds have “underdeveloped” wings, paedomorphic processes may initiate at least some flight losses (see ref. 30). However, this idea is difficult to assess in long-flightless wild birds. Runners and Hybrids, though, represent much earlier stages of flight loss and show that the developmental assembly of “flightless” features can be somewhat surprising (*SI Appendix*, Table S7).

#### Relative reductions in wing investment and performance in Hybrids and especially Runners are driven by increases in body size (peramorphosis) rather than reductions in wing size (paedomorphosis).

In Runners and to some extent Hybrids, the flight apparatus is proportionally smaller than that of Mallards, as predicted for birds with reduced flight. However, proportionally smaller wings were due to increases in body size (Fig. 1) rather than reductions in feather area or forelimb muscle mass, which were surprisingly conserved (Fig. 2). Proportionally smaller wings nevertheless manifested as declines in wing performance, which was lower in birds with higher wing loading and lower relative muscle mass (Fig. 3). Thus, Mallards had the best wing performance, Runners had limited flight capacity but probably more than a bird with paedomorphic wings, and Hybrids, as the transitional state, were generally intermediate. Given that Ostriches (*Struthio* spp.) also retain large wings with embryonic growth rates similar to those of volant birds (30), these findings suggest that, in at least some birds, initial declines in wing investment and performance are actually driven by increases in body size (peramorphosis) rather than reductions in wing size (paedomorphosis).

#### Legs are labile: compared to Mallards, Runners—and presumably Hybrids—have proportionally smaller legs as juveniles but larger legs as adults (peramorphosis).

Flightless birds are clearly leg-dependent, and many have large hind limbs (6). At what point of flight loss do legs get larger? We found that, unlike wings, legs are not conserved. In all three groups, an initial period of leg growth and performance improvement was followed by leg muscle resorption and declines in performance as wing muscles grew (Figs. 4 and 5). However, these changes were most extreme in Mallards, which had proportionally larger leg muscles as juveniles but smaller leg muscles as adults, because Hybrids and especially Runners regained leg muscle after wing growth slowed. These differences are likely related to shifts in resource allocation: Hybrids and Runners invest proportionally more resources into body growth as juveniles and proportionally less into wings as adults. Thus, legs are labile, and the increases in leg investment that accompany declines in relative wing investment are associated with leg peramorphosis. Overall, peramorphosis yielded wingier Mallards, intermediate Hybrids, and leggier Runners (*SI Appendix*, Fig. S7), with Mallards being good fliers and Hybrids being fast runners.

### Conditions and Behaviors that Facilitate Flight Loss and Associated Shifts in Investment.

In wild birds, flightlessness is associated with locations where wings are not needed to escape predators or migrate. Runners clearly do not need to migrate because they were domesticated in regions with mild climates and are now fed in areas with cold winters. Based on historical records (*SI Appendix*, *Text S2*) and our own data, three factors have likely contributed to the near-loss of flight in Runners and provide additional insight into conditions and behaviors that may facilitate flight reduction.

#### Protected juveniles: shifts in investment and increases in body size.

Runner Ducks have been prized for their high egg production. Yet they generally do not incubate their own eggs. Given that incubating females typically stop laying (31), high egg production may have required egg removal and artificial incubation. After hatching, juveniles were reared by humans. This protection may have freed young Runners to invest less in their legs and leg-based escape behaviors (Figs. 4 and 5), and more in body growth (Fig. 1). Wild flightless birds are also often larger than their volant relatives (6). Similarly, passerine nestlings allocate more to body growth when predation is lower (32), and altricial birds reared in nests tend to invest relatively less in their legs than precocial birds that must navigate their environment soon after hatching (33). Thus, humans might have mimicked low-predator habitats or guarded nests, and such protection may facilitate a shift in investment from locomotor structures to body size—perhaps especially in juveniles, when body growth is occurring.

#### Efficiency(?) over escape; escape over long-distance flight?

As adults, Runner Ducks were—and often still are—herded to fields for eating pests and fertilizing crops. Several aspects of their anatomy may be adaptations to this terrestrial, ambulatory lifestyle. First, reduced dependence on aquatic habitats might have improved terrestrial locomotion, because specializations for swimming likely compromise walking. For example, compared to terrestrial birds, swimming birds may have shorter legs (34), less efficient waddling gaits (35), and medially oriented feet during stance (36). Longer, more muscular legs (Fig. 4) that are better aligned with walking direction (preliminary data) might have evolved as Runners became less aquatic, long-distance walkers. Similarly, large feet—though useful for swimming—might have reduced sinking in muddy fields, like snowshoes (37). Finally, shifting from a more horizontal, Mallard-like posture to the upright, penguin-like posture that Runners are known for may have enhanced terrestrial efficiency.

For example, in Whistling Ducks (*Dendrocygna* spp.), more terrestrial species also tend to walk with more vertical postures (38). Studies suggest that this 1) centers body mass more over the hip, reducing hip torque, and 2) permits greater hip extension, which could 3) allow for a more elastic (bouncier) gait on extended limbs (39–41). Such changes could improve terrestrial efficiency. However, they would also shift gaits away from the crouched, compliant “grounded running” of most avians (39, 42). Crouched, compliant gaits—while energetically costly—allow for prolonged foot contact and force generation, resulting in lower peak forces and greater stability (40, 43–46). Given that Runners probably have a less compliant gait with more extended limbs and shorter foot contact times than Mallards (47, 48), they may be more efficient, but also less stable at high speeds. This could be exacerbated by their large feet, which often appeared awkward during running.

Runner Ducks, then, might be adapted for energy-efficient, rather than fast, terrestrial locomotion and may have relied less on speed and more on protection from their human attendants. This would reiterate that birds might prioritize energy conservation over escape performance in areas with sufficiently low predation (12). Hybrids, in contrast, may be more like island birds that experience lower but persistent predation (49). Although their flight capability is reduced and they probably cannot fly very far, their leg performance is higher (Fig. 5), and this may allow them to maintain high escape performance. Indeed, leaping into flight is a common escape tactic, and Hybrid takeoff velocities (Fig. 6) are, if anything, faster than those of Mallards. This would reiterate that whole-body performance often depends on the coordinated use of wings and legs, whose relative contributions vary by behavior and habitat.

#### Wing-leg coordination and shifts along a flightless-to-flying spectrum.

Although often described as flightless, Runner Ducks retain some flight ability because their wing muscles and feathers are approximately conserved (Figs. 2 and 3). This conservation may indicate that wings initially change more slowly than bodies and legs as flight is lost. Though the flight apparatus can change rapidly under artificial selection (50, 51), in wild flightless lineages, flight feathers do appear to change more slowly than body mass and the musculoskeletal system (52). This may be because feathers are comparatively less metabolically expensive to maintain and have complex development, limited plasticity, and/or low genetic diversity (50, 52)—possibly due to strong selection for flight. Given that Runners have not been intentionally selected for feather structure, this may explain why their feathers are conserved.

But why do Runners have conserved flight muscles if maintaining them is energetically costly (12)? One possibility is that because Runners are at an earlier stage of flight loss, both feathers and muscles are still constrained by a long history of selection for flight. Yet, Runners were likely domesticated over 1,000 y ago. Given how quickly the flight apparatus can change under artificial selection, this should be sufficient time for at least some changes to occur and is probably not the full explanation. A second possibility is that Runners have been artificially selected for meat production (though this does not seem to have been a primary consideration), or have access to more food than wild birds, alleviating energetic burdens. However, we offer a third and perhaps underappreciated possibility: that Runners may have retained their wings at least partially because their wings are still valuable, especially when engaged with the legs. We have observed our birds using their wings and legs cooperatively to swim, jump for food, or fly short distances after leaping away from a threat—all of which would presumably be advantageous to a free-range bird. Such “transitional” behaviors are known to play a crucial role during the development of flight by bridging leg-based behaviors of young birds with wing-based behaviors of adults (9, 53–55). Perhaps, then, wings play a reverse role during evolutionary reductions in flight, bridging wing-based behaviors of ancestors with leg-based behaviors of descendants. Birds that, like ducks, are flightless for long periods as juveniles and during wing molts would be particularly adept at traversing this bridge (13–16).

## Conclusions

Flying Mallards, semi-flight-capable Hybrids, and flightless Runner Ducks collectively span a wide range of flight capacities. Future work will examine detailed relationships between adult anatomy and performance to better understand wings versus legs in the avian *bauplan*. Here, however, our results show that Runners share many features with wild flightless birds, that Hybrids are intermediate, and that the assembly of these intermediate and flightless features is surprisingly driven by increases in body and leg size (peramorphosis) rather than reductions in the flight apparatus (paedomorphosis). This contrast between wing conservation and body and leg peramorphosis suggests that during flight loss in at least some birds, body and leg size initially change more rapidly than wings, with the flight apparatus becoming proportionally smaller before its components are reduced. In wild birds, such reductions usually occur in safe habitats with mild climates, where wings are not needed for migration or escape. Our results, coupled with the history of Runner Ducks, underscore the importance of both food and protection during flight loss, but particularly for growing juveniles. Our data also reiterate that birds might prioritize growth or energy conservation over escape performance when predation is sufficiently low, and escape over long-distance flight if predation is reduced but persistent. Given that juveniles with incipient flight and adults with reduced flight both engaged their wings and legs cooperatively, wing-leg coordination likely facilitates these shifts along a flightless-to-flying spectrum, exemplifying how animals can use transitional behaviors as specialized structures are gained or lost.

## Materials and Methods

Ducks were raised and trained following IACUC-approved protocols (*SI Appendix*, *Text S4*).

### Anatomical Measurements.

Anatomical measurements were taken regularly throughout ontogeny. Birds were initially measured 2 to 3 times per week, but less frequently as growth slowed. 5 to 6 Runners, 3 to 6 Mallards, and 6 Hybrids were measured each time, using different birds and, as much as possible, an even mix of males and females (all breeds), colors (Runners and Hybrids), and reciprocal cross types (Hybrids). Subsets of birds were killed humanely at regular intervals for dissection, via an overdose of Isoflurane anesthetic.

Body mass, wing or feather area and loading, foot area and loading, muscle masses, and bone lengths were measured as in previous works (e.g., refs. 9 and 18) (*SI Appendix*, Text S5).

### Wing and Leg Performance.

#### Locomotor behaviors.

Four behaviors relevant to ducks were filmed and analyzed throughout ontogeny: running, swimming, controlled flapping descent (CFD), and jumping or vertical takeoff (VT) (*SI Appendix*, Text S4). Filming coincided with anatomical measurements, so that the same birds were measured for anatomy and performance. All behaviors were filmed using a Fastec camera recording at 250 to 400 fps. For digitizing purposes, birds were marked on the head ± breast with tape. Different birds were filmed each session to maintain high motivation, and each bird was filmed multiple times per behavior. All trials were qualitatively scored based on the bird’s effort, and filming continued, with breaks, until 2 (CFD) or 3 (other behaviors) “good” or “very good” trials were recorded. If a bird seemed unmotivated, it was replaced. All birds were rewarded with treats after completing a trial.

#### Digitizing and performance metrics.

For each trial, the tape marker was digitized using DLTdv8a (56). Digitized points were calibrated and then imported into IgorPro (57), where velocity and acceleration were calculated. First, positional data were graphed and smoothed (“box” smoothing, equivalent to a low-pass filter), although this did not visibly alter the data. Smoothed positional data were differentiated and graphed to determine velocity, which was then smoothed, differentiated, and graphed to determine acceleration, which was also smoothed.

For running and swimming, maximal velocities—based on velocity graphs—were reported for each trial, after confirming that the value was not due to a spike caused by digitizing error or a bird leaping or flap-running. The fastest velocity achieved by a given bird at a given age was reported. Since birds ran on a straight trackway, running velocities were calculated along a single direction of movement (e.g., x velocity). In contrast, birds often did not swim straight across the pool. We therefore primarily filmed them in dorsal view and calculated velocity in the horizontal plane [e.g., (x^2^ + y^2^)^0.5^ velocity].

For CFD and VT, wing performance was defined as vertical aerodynamic force production, in multiples of body weight [after (9)]:

[1] $$\text{Force}\;(\text{multiples}\;\text{body}\;\text{wt})=\frac{{\mathrm{ma}}_{w}}{m\left|g\right|}=\frac{a_{n}-g}{\left|g\right|},$$

where *m* is the mass of the bird, *a_w_* is (upward) acceleration due to aerodynamic force generated by wing flapping (m/s^2^), *g* is (downward) acceleration due to gravity (−9.81 m/s^2^), and *a_n_* is the net acceleration (*a_w_* + *g*) calculated from video analysis (above). Force production was averaged over 3 wingbeats (CFD) or 1 to 3 wingbeats after takeoff (VT), depending on the ability of the bird, and the best set of consecutive wingbeats was reported. For VT, this metric indicates a bird’s ability to support its body weight and sustain flight. However, some birds, like galliforms, are specialized for brief but rapid bursts of flight (9, 58). Thus, peak vertical velocity after takeoff was also reported. Finally, because takeoff is initiated by the legs, we also analyzed vertical velocity during this grounded phase of jumping.

### Ontogenetic Trends.

#### Anatomical growth curves and age class comparisons.

Anatomical measurements were plotted against age to assess ontogenetic trends. Logistic growth curves, commonly used to describe growth in birds (59), were fitted to anatomical data using nonlinear regression models (“nls” function) in R (60). For wing area, curves were fit up to 83 to 88 d, because areas appeared to decline slightly afterward, possibly due to shrinkage of feather follicles and papillae during the rest phase (61). Model parameters were checked for statistical significance, and each growth curve’s first derivative was used to compare growth rates. For data that could not be fit with a growth curve, trends were visualized using smoothing splines or lines between age-class averages. One-way ANOVA + Tukey post hoc tests (“aov” and “TukeyHSD” functions in R) were used to compare Mallards, Runners, and Hybrids at a specific age (e.g., juvenile, adult). Anatomical declines ± regains were assessed using *t* tests to compare younger and older birds.

#### Anatomical scaling.

To assess how anatomical structures changed with increasing body mass, data were common log transformed and plotted against log body mass, then fitted with linear regression models (“lm” function in R) with parameters assessed for statistical significance. To determine whether structures were growing isometrically (with a constant relative size) or with positive or negative allometry (with relative size increasing or decreasing, respectively), slopes were compared to expectations under geometric similarity (uniform scaling = isometry). In most cases, younger and older birds had to be fitted with separate models due to clear shifts in scaling. Even within some of these subgroups, data were not perfectly linear. However, we did not further subdivide if there was no obvious place to do so, because our goal was to examine overall patterns of growth and how structures were growing on average. Once growth patterns were established, we used ANCOVA (“aov” or “lm” function in R) to determine whether slopes differed across Mallards, Runners, and Hybrids. If slopes looked similar and did not differ significantly, we then repeated the test to see whether intercepts differed when slopes were assumed identical (no interaction between variable and breed).

#### Performance.

Performance metrics were similarly plotted against age, and ontogenetic trends were visualized by lines passing through age-class averages. Differences in performance between Mallards, Runners, and Hybrids at a specific age (e.g., juvenile, adult) were assessed using one-way ANOVA + Tukey post hoc tests. Performance declines were assessed using *t* tests to compare younger and older birds.

## Supplementary Material

Appendix 01 (PDF)

Movie S1. Controlled flapping descent (CFD) in a 6 day old Mallard.

Movie S2. Controlled flapping descent (CFD) in an adult Hybrid.

Movie S3. Vertical takeoff (VT) in a 14 day old Hybrid.

Movie S4. Vertical takeoff (VT) in an adult Runner.

Movie S5. Running in a 24 day old Hybrid.

Movie S6. Steaming in a 52 day old Hybrid.

## Data Availability

All study data are included in the article and/or supporting information.
