# Supplementary material for: A transition toward flightlessness in Mallards, Indian Runner Ducks, and their hybrid offspring
Source: Proc Natl Acad Sci U S A. 2026 Jul 20;123(30):e2534729123. doi: 10.1073/pnas.2534729123 (PMC13416651; doi:10.1073/pnas.2534729123)
Supplement: Supplementary file 1 — Appendix 01 (PDF) [file pnas.2534729123.sapp.pdf]

PNAS

Supporting Information for
A Transition Toward Flightlessness in Mallards, Indian Runner Ducks, and their Hybrid Offspring

Ashley M. Heers, Willa Coultley, and Sandy A. Gregorio

corresponding author: Ashley M. Heers
aheers@natsci.claremont.edu

This PDF file includes:

Supporting texts S1-S5
Figures S1-S7
Tables S1-S7
Legends for Movies S1-S6
SI References

Other supporting materials for this manuscript include the following:

Movies S1-S6

Table of Contents

Text S1. Ontogenetic and evolutionary acquisitions of flight 3

Text S2: History of the Indian Runner Duck 4

Text S3: Jumping performance 6

Text S4: Animal care and training 7

Text S5: Anatomical measurements 9

Figure S1: General appearance 11

Figure S2: Feather development 12

Figure S3: Wing bone proportions 13

Figure S4: Vertical takeoff score 14

Figure S5: Leg bone proportions 15

Figure S6: Swimming 16

Figure S7: Tradeoffs between wings and legs 17

Table S1: Results of statistical tests 19

Table S2: Body mass 23

Table S3: Wing area and loading 24

Table S4: Bone length 25

Table S5: Muscle mass 26

Table S6: Foot area and loading 27

Table S7: Allometry 28

Movie legends 29

References 30

Text S1: Ontogenetic and evolutionary acquisitions of flight

Rudimentary versions of the avian flight apparatus are found both in the theropod-avian fossil record (reviewed in (1, 2)) and among extant developing birds, which typically hatch with small or non-existent wings, small muscles, and underdeveloped skeletons (3–6). A variety of new techniques are providing insight into fossils (reviewed in (7)), and studies on baby birds offer real-world examples of how rudimentary structures are used. These studies show that although many young birds do not yet fly, their "protowings" can nevertheless play an important role in locomotion. Fledglings may flap their rudimentary wings to improve foot traction while scaling steep surfaces (8), slow aerial descents (9), extend jumps (10), and/or swim underwater (11) and steam across water (12), depending on environmental context. This cooperative use of wings and legs allows developing birds to smoothly transition from leg-based terrestrial or aquatic behaviors to wing-based aerial behaviors (5, 13). For example, the development of the flight apparatus and attendant improvements in aerodynamic performance enable young galliforms to flap-run up progressively steeper inclines, increase jump height, and eventually achieve flight (8, 10, 14). Juvenile birds thereby demonstrate that incipient flight apparatuses are immediately functional and can play an important role in the acquisition of flight. Collectively, these studies of fossils and developing birds are providing powerful insight into the ontogenetic and evolutionary construction of the avian body plan and how flight is/was acquired.

Text S2: History of the Indian Runner Duck: a summary of (15–21)

Though the early history of Indian Runner Ducks is convoluted, and much remains unknown, accounts nevertheless agree that Runner Ducks are highly active, terrestrial foragers. Records indicate that Runner Ducks were imported into Great Britain multiple times in the early 1800s and possibly sooner, apparently at least once by a sea captain who traded in “the Indies”. From England, the birds were then imported into the United States. The account of the sea captain, combined with a “peculiar running gait”, gave rise to the name “Indian Runners”. British and U.S. farmers prized these birds as “splendid” egg layers – maturing quickly and “producing an egg almost daily”, and for pest management – “they will follow the plough and eagerly devour the worms and grubs”, “they require very little food to be given them as they search for it themselves”, and “low fences will hold them”. Unlike other domesticated ducks, Runners were not bred specifically for meat production, although they were eaten (“I cannot say so much in their favor for the table as they are a small, though very sweet food”).

By 1900, however, the Runner Duck breed was a “mixed and confusing state of affairs” in both Britain and the United States. Dilution of the early stock appears to have stemmed from a combination of casual outbreeding with other domesticated and wild ducks, as well as conflicting attempts by fanciers to breed birds with specific colors for exhibition and by farmers to favor birds with high egg production and foraging capacity. All of this was confounded by a history of repeated imports from uncertain locations without knowing the Runner Duck’s origins. Around this time, the Indian Runner Duck Club was formed in Britain, and, along with U.S. breeders, it began establishing standards for the breed. However, there was disagreement over what the “true” form was, with fanciers favoring various colors and farmers favoring utility. Several breeders and club members thus set out to locate the birds’ place of origin.

Finding the original source of Indian Runner Ducks proved to be an incredibly challenging endeavor. After discovering some Runner-like ducks and skins labeled “Darwin’s Penguins” in a museum, and exchanging letters with Alfred Russel Wallace – who had described “Penguin Ducks” in one of his accounts and had sent them to Darwin, ducks matching original descriptions were eventually tracked down and imported from several islands of the Malay Archipelago (Lombok, Bali, Java), thereby saving and rejuvenating the bloodline. Though “true” Runner Ducks could only be found in rural areas, they had apparently been in the region for some time; reliefs resembling Runner Ducks were reported on ancient temples in Java that are possibly over one to two thousand years old.

Based on these accounts, it is therefore likely that Runner Ducks originated somewhere in the Malay Archipelago and adjacent Southeast Asia, possibly over a thousand years ago. Duck herding has been a tradition in this area for centuries, where ducks are traditionally kept as large, outdoor flocks. After their eggs are collected in the morning, they are led out to fields or rice paddies with an attendant, where they traverse long distances while actively foraging for insects, snails, worms, fallen grains, and, in some areas, small fish. Farmers report that ducks thrive in areas where other stock do not and till the soil as they search for food, help with weed and pest control, and fertilize plants with their feces (e.g., rice-duck farming) or even feed fish with their feces (duck-cum-fish farming). At the end of the day, the ducks are returned home to outdoor enclosures.

The earliest reports of “Runner Ducks” in the Malay Archipelago described them as having long legs, walking rather than waddling – often through shallow water in rice paddies, and as being highly active foragers that rarely use their wings, which were noticeably small for

their body size. They were easily distinguished from other ducks by their upright posture (~70° above horizontal), and some naturalists referred to them as “Penguin Ducks” for this reason. Early farmers observed that when predators were spotted, their ducks would hide beneath vegetation, and it was suspected that their native, “mud”-colored feathers aided in camouflage. Though Runners were not a very “broody” duck, they were valued enough that their eggs were incubated and juveniles were raised for sale to farmers, who then took the birds for egg laying and field foraging. Many birds were walked for very long distances before reaching the market, such that only the best walkers and layers were sold. In short, Runner Ducks in the Malay Archipelago and surrounding regions were likely highly active and valued long-distance foragers and fertilizers with an erect gait and posture, high egg production, and little propensity for flight.

Following the near loss of the breed in the early 1900s, the Indian Runner Duck Club and other breeders took care to preserve these features in their newly imported stock from the Malay Archipelago. Standards were revised to maintain the breed's purity, although breeders continued to experiment with feather coloration, resulting in many new color varieties. Today, different countries have slightly different Standards for Runners. For example, the American Standards and English Standards differ in coloring (8 recognized colors in the United States versus 14 in Britain). In Britain, birds with more upright postures are favored for exhibition, whereas in the United States, birds are more commonly valued for egg production and foraging, and posture is more variable (45-75° above horizontal). Although Runner Ducks are popular show birds and sometimes kept as pets or for training herding dogs, they are also still widely used around the world as egg layers and for organic pest control and fertilization in backyards, farms, rice paddies, and vineyards. In fact, current descriptions of the breed largely mirror original ones, depicting Runners as slender birds with an upright posture and a “smooth running gait”, as “outstanding egg layers”, “active” and “eager” foragers that traverse large areas and are “more at home on land” than other breeds, “wonderful pest controllers”, and unable to sustain flight.

Though domesticated, in many respects, Runner Ducks are thus still free-range: they remain highly active, outdoor animals that, in many areas, forage for much of their food over long distances.

Text S3: Jumping performance

Originally, we intended to quantify leg force production during jumping (i.e., jump component of vertical takeoff). However, we were a new lab and did not have a force plate set up at the time. Later, when we checked leg force measured by a force plate with leg force calculated from high-speed video, we found that video calculations greatly overestimated leg force production, at least in older birds, even when we examined leg force before wing downstroke began. We believe that these discrepancies are due to body rotation and extension. Younger birds sometimes displayed a countermovement during jumping, but often did not. In contrast, older birds nearly always used an exaggerated countermovement and rapidly rotated their bodies from nearly parallel with the ground to nearly vertical. These rapid rotations occurred prior to leg extension and coincided with the highest acceleration levels in videos. This challenge in measuring leg forces may have been circumvented if the hip had been marked rather than the head and chest, but hip position is challenging to visualize, and we did not attempt to do so. Even so, “jump force” was observed to increase, peak, and then decline similarly to running performance, after which it plateaued or increased as older birds began employing exaggerated countermovements.

Text S4: Animal care & training

1. Animal Care

45 Runner Ducks and 15 Mallards were purchased from commercial hatcheries. Ducks were housed in Animal Care Facilities at California State University, Los Angeles, for ~6 months, following IACUC-approved protocols (IACUC 1020-01). Birds were divided into multiple enclosures (just Mallards, just Runners, or a mix of both, depending on bird personalities). Enclosures were identical across groups, with shavings (which were better than mats or hay) on a flat floor and multiple food and watering stations, but no pools or elevated surfaces, except for a sink. A large pool was maintained in the lab, and birds were allowed to swim in the pool 1-2 times per week. All birds were given heat lamps as needed, and food and water *ad libitum*. Juveniles were fed Mazuri Waterfowl Starter food supplemented with mealworms, peas, first-cut alfalfa, nutritional yeast, oats, hard-boiled egg yolk, and grit. Adults were transitioned to Mazuri Waterfowl Maintenance food, with many of the same supplements, and given access to oyster shells.

A subset of Runner Ducks and Mallards was adopted out and allowed to breed over winter, to get reciprocal crosses. Eggs were collected each morning, marked, and then incubated for ~29 days. We used two Manna Pro Harris Farms Nurture Right 360 incubators. Incubator temperature and egg rolling were automatically controlled, but water was added several times daily to maintain the recommended humidity. Below are the settings we used:

- Temperature = 99.5° for days 1-25, 98.5° for days 26-29
- Hourly egg rolling for days 1-25, no egg rolling for days 26-29
- Humidity = 57-62% (average ~60%) for days 1-25, ~75% for days 26-29

Aside from a few unfertilized eggs, all eggs except for one hatched. The incubators were offset by 7 days, resulting in two groups of hybrids hatched one week apart.

Although ducks were raised across two years (Mallards and Runners the first year; Hybrids and some additional Mallards and Runners the second year), their hatch dates were very similar (late spring), so seasonal influences should not have differed.

2. Animal Training and Trials

2.1 Running

Running was filmed in lateral view on a 15-foot trackway lined with rubber mats and walled by plexiglass. Birds were encouraged to run toward an area with other ducks by throwing treats to be chased, releasing a “leader” and then “followers”, and/or allowing birds to “escape” from a cat toy. Running in groups of 2 to 3 seemed to work best. As birds grew older, they sometimes flapped their wings and appeared to leap rather than run; these trials were analyzed separately. Mallard data was supplemented by data from (12) (up to 60 days).

2.2 Swimming

Swimming trials were mainly filmed in dorsal view, in a 10-foot pool. Ducklings were encouraged to swim after a “leader” bird or toward clutch mates. As birds grew older, they were additionally startled into swimming with a cat toy. Adults swam the fastest while playing (racing around the pool). Trials were classified as “swimming” if the wings did not touch the water, “steaming” if the wings were used like oars, or “water launch” if the bird launched itself up and out of the water. Mallard data was supplemented by data from (12) (up to 60 days).

2.3 Controlled Flapping Descent

To quantify controlled flapping descent, birds were held in a horizontal body position with their wings folded, then released to descend to a pillowed landing 3-9 feet below. This drop distance was increased as birds improved, so that at least 4-5 vigorous wingbeats were recorded. Body rotations just after release and prior to landing were excluded from analyses.

2.4 Vertical Takeoff

To quantify vertical takeoff or ascending flight, ducks were encouraged to jump $\pm$ fly ~vertically from a small platform up to an elevated platform with other ducks. This upper platform was positioned relatively close for the first trials of the day. When birds were successful, it was slightly raised, and trials continued. This process was repeated ~3-4 times, until a bird was barely able to reach the upper platform. Pillows were placed below to prevent injury in the event of a fall, but falls were extremely rare because we gave boosts as needed to help birds maintain confidence. This was important for juveniles and all Runner Ducks.

Text S5: Anatomical measurements

1. Body Mass

Body mass (g) was measured with an OHAUS scale, with birds constrained by a box.

2. Wing area and loading

To photograph the wings, each bird's wing was gently stretched over a table to its fullest extent. Tertial feathers were separated from the body by felt. The wing was then photographed in dorsal view, using a Nikon or Fastec camera. Images were imported into ImageJ (22) and calibrated using rulers for scale. Each wing was then manually outlined using the "polygon" tool, and measured (cm^2). Finally, wing area was doubled, and body mass was divided by this two-wing area to calculate wing loading (g/cm^2).

3. Foot area and loading

To photograph the feet, one foot of each bird was pressed gently over a sheet of glass, with the toes fully spread. The foot was then photographed in ventral view, using a Fastec camera. Images were again imported into ImageJ and calibrated. The foot was then manually outlined, excluding claws, and measured (cm^2). Foot loading was calculated by dividing body mass by the area of a single foot (g/cm^2), since a single foot often propels the body.

4. Dissections: feather areas, muscle masses, and bone lengths

All measurements taken on live birds were also taken on specimens prior to dissection. In addition, primary and secondary feathers were removed at their point of contact with the skin, flattened by glass, and photographed. Photographs were imported into ImageJ, and individual feather areas were measured using the "polygon" tool (cm^2). After feather removal, wing and leg muscles were removed from both sides of the body and weighed on an OHAUS scale (mg-g).

Wing muscles (i.e., all muscles crossing a wing joint) were dissected first. All muscles were identified, photographed, and then removed and weighed, in approximately the following order: pectoralis, supracoracoideus, propatagialis brevis, deltoideus major, scapulotriceps, latissimus dorsi, humerotriceps, biceps brachii, scapulohumeralis caudalis, subscapularis, coracobrachialis posterior, subcoracoideus, coracobrachialis anterior, deltoideus minor, and antebrachial muscles. The antebrachial muscles (extensor metacarpi radialis, extensor pollicis longus, extensor carpi ulnaris, extensor digitorum communis, anconeus, supinator, pronator sublimis, flexor carpi ulnaris, flexor digitorum sublimis, flexor digitorum profundus, pronator profundus, entepicondyloulnaris, brachialis, ulnimetacarpalis ventralis, and extensor indicis longus) were weighed as a single group except in two adults, where they were removed and weighed individually.

Leg muscles (i.e., all muscles crossing a leg joint; names after (23)) were dissected second, in approximately the following order: iliotibialis lateralis, iliotibialis cranialis, flexor cruris lateralis pars pelvica, iliofibularis, caudofemoralis pars caudalis, caudofemoralis pars pelvica, iliotrochantericus, ischiofemoralis, femorotibialis externus, femorotibialis medius, flexor cruris medialis, puboischiofemoralis lateralis, puboischiofemoralis medialis, femorotibialis internus, ischiofemoralis, gastrocnemius (externus / lateralis, intermedius, internus / medialis), fibularis longus, and tibialis cranialis. The remaining shank muscles were removed and classified as flexors or extensors, and then weighed together.

Following muscle removal, the lengths of the humerus, ulna, carpometacarpus, femur, tibiotarsus, and tarsometatarsus were measured using digital calipers (mm). Mallard bone and muscle data were supplemented by data from (12) and muscles were scaled to match, since not all muscles were included in (12).

Figure S1: General appearance

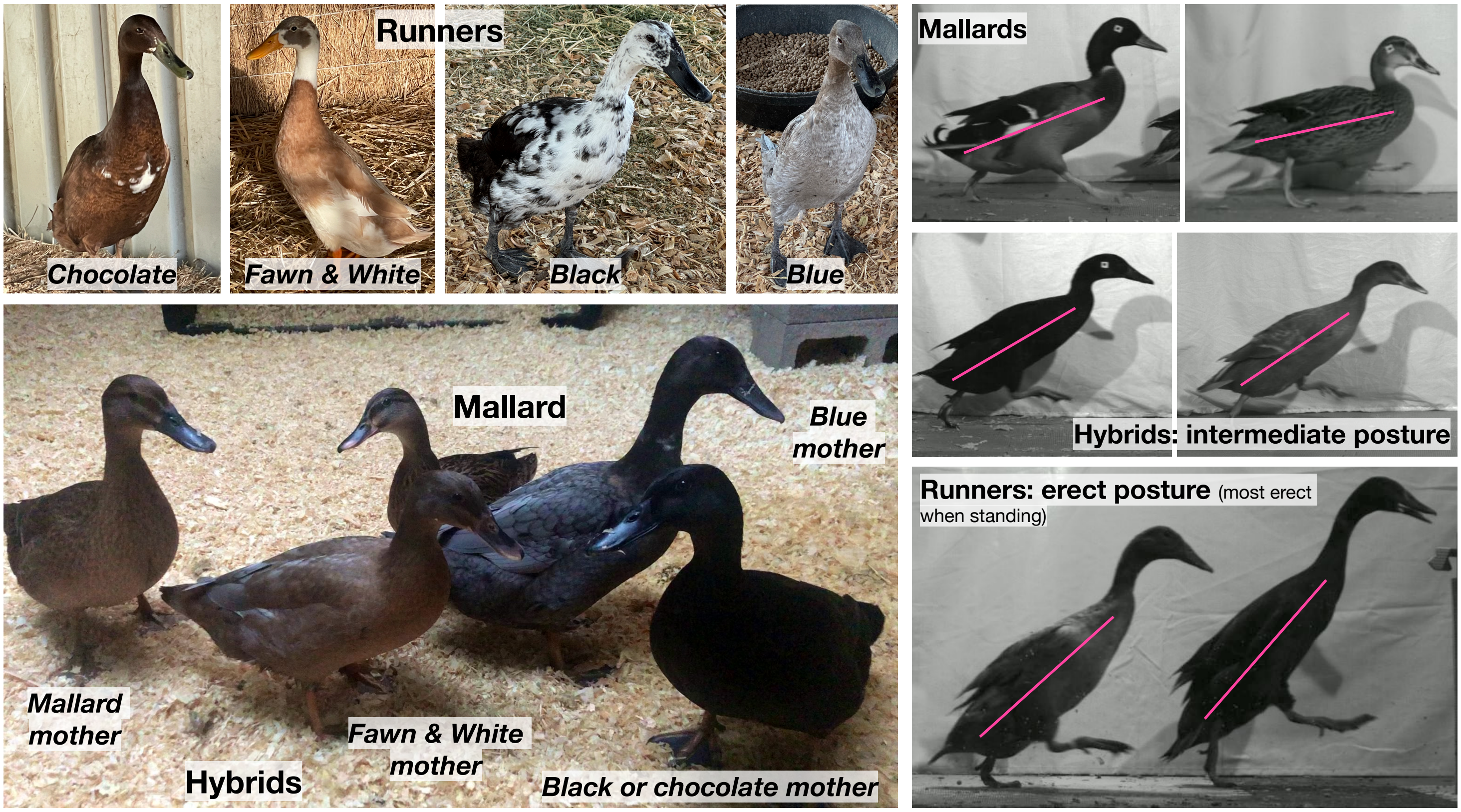

Variations in color (left) and posture (right). Black and chocolate female Runners and Hybrids were entirely dark colored their first year; white feathers appeared with successive feather molts. Hybrids had unique feather coloration that more closely resembled that of their female parent.

Figure S2: Feather development

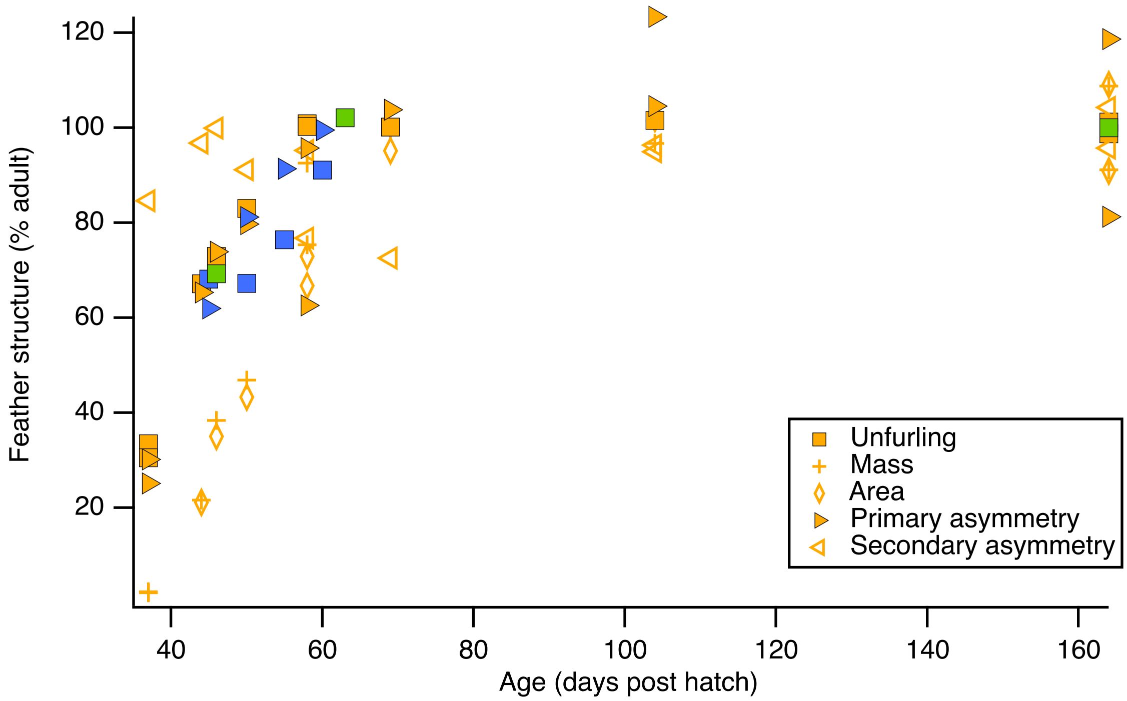

Wing feathers begin to emerge at 17-19 days (like other waterfowl (24); Table S3), and become clearly visible at ~ 30-34 days. Runners (orange), Mallards (blue), and Hybrids (green) then show similar patterns of feather development: Mallards and Hybrids (fewer samples available) fall within the range measured for Runners.

Figure S3: Wing bone proportions

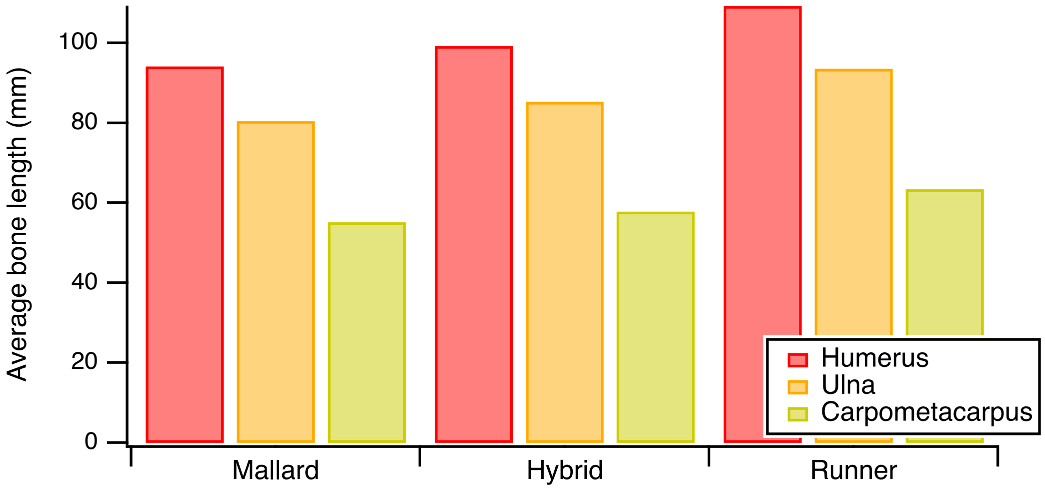

The longer forelimbs of Hybrids and Runners appear to result from increases in length across all limb elements.

Figure S4: Vertical takeoff score

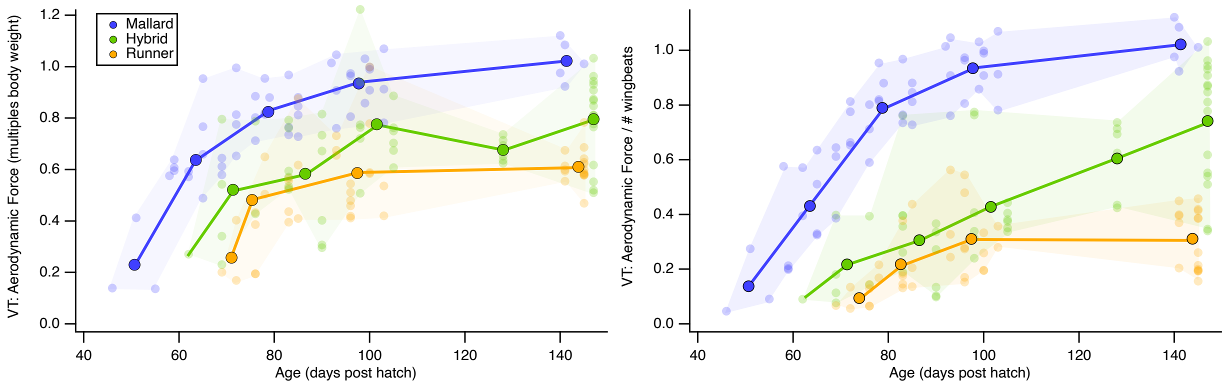

Quantifying wing performance during vertical takeoff in birds with limited flight capacity is challenging. Initially, performance was quantified as aerodynamic force production (left). However, Runners and some Hybrids occasionally gave a “burst” of high wing performance during one wingbeat, and then were unable to sustain flight afterward. This “inflated” their performance compared to birds that performed more steadily over multiple wingbeats. In addition, there was a period of time when Hybrids and Runners performed well for one wingbeat but couldn’t quite complete two. When they finally transitioned to two wingbeats, their performance dropped compared to the burst they had during one wingbeat (see drop in Hybrid performance after 100 days). We attempted to account for these challenges by dividing force production by the number of wingbeats (right). However, this altered the Hybrids' overall ontogenetic trajectory (i.e., the general trend was driven more by the number of wingbeats than by aerodynamic force production). We therefore used an aerodynamic force production “score” (Fig. 3D) that was a compromise between the two graphs above:

VT score = (aerodynamic force / BW)(# wingbeats + 3)/6

This penalized birds slightly for completing fewer than three wingbeats but maintained the general ontogenetic patterns.

Figure S5: Leg bone proportions

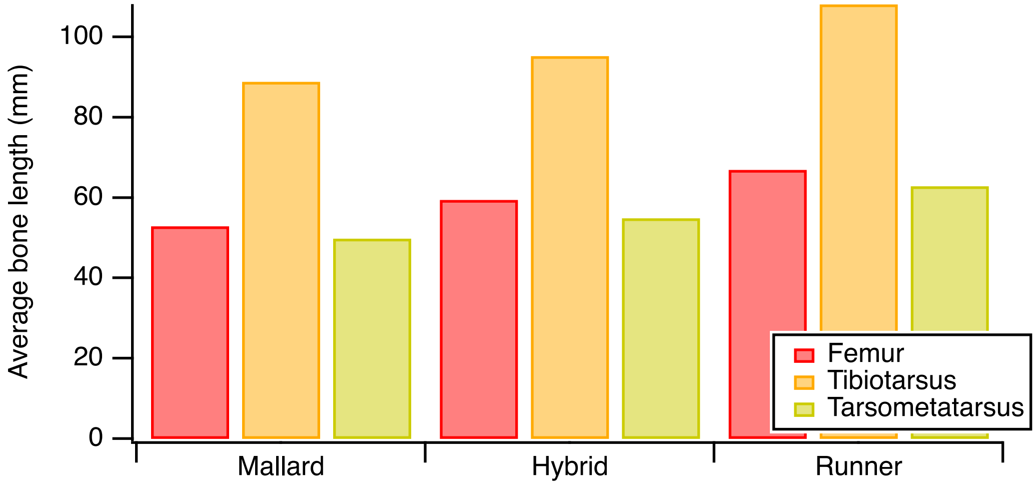

The longer hind limbs of Hybrids and Runners appear to result from increases in length across all limb elements.

Figure S6: Swimming

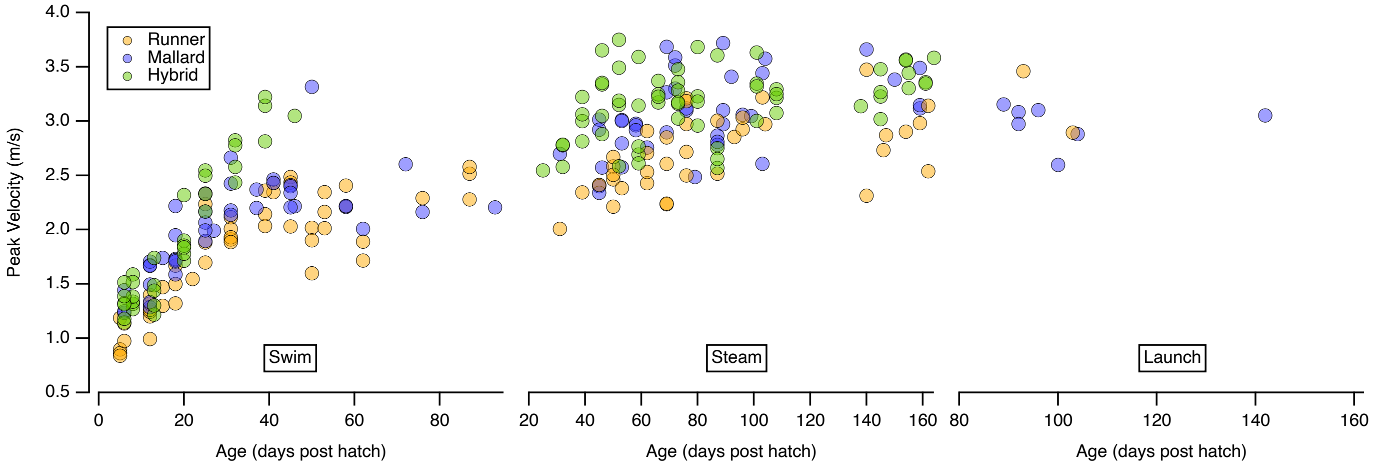

When swimming is analyzed without wing contributions, performance stops improving around 45 days. Similarly, Mallards show a brief performance dip between 35 and 53 days (Fig. 6A), perhaps due to their steep decline in leg muscle before wing contributions are significant. A similar, temporary dip may occur during vertical takeoff (Fig. 6B).

Figure S7. Tradeoffs between wings and legs

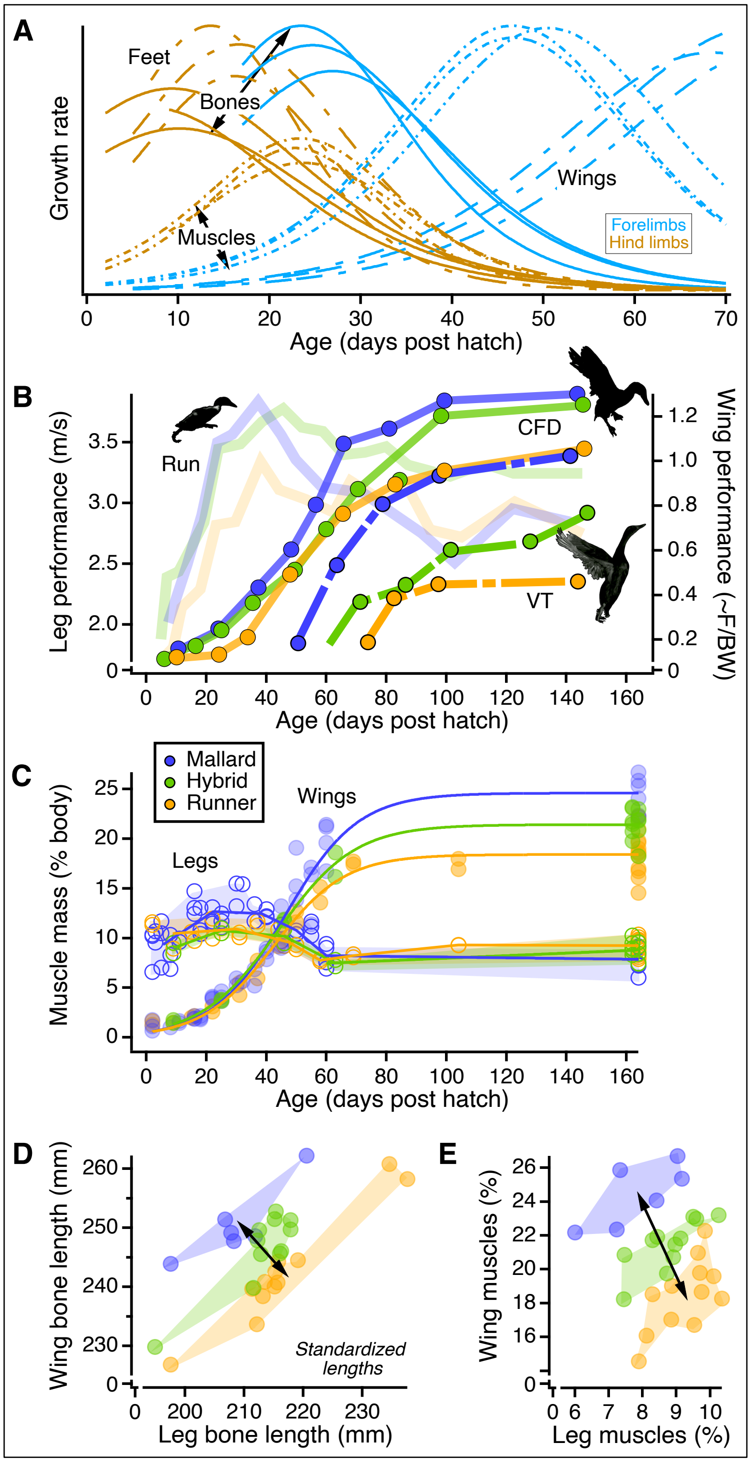

(A) When wings and legs are considered together, leg development clearly precedes wing development in all three groups: feet grow before wings, leg bones before wing bones, and leg muscles before wing muscles. Within the wings, bones develop first, then muscles, and finally feathers. Similar patterns have been observed in other birds with long wings, potentially because bones grow more slowly and take longer to reach full size (25). These anatomical offsets are accompanied by (B) offsets in performance, with legs peaking before wings. As ducks transition to varying degrees of flight capability, (C) leg

investment and (B) performance decline, especially in Mallards, which have proportionally (D) longer and (E) more muscular wings as adults, but shorter and less muscular legs. Such patterns align with previous work suggesting that wing investment and performance can increase at the expense of leg investment and performance, or vice versa, during ontogeny and across species (10).

Growth rates (A), performance (B), and muscle development (C) or adult muscle mass (E) from previous figures. Adult bone lengths in (D) scaled to average body size of all three groups.

Table S1: Results of statistical tests
(bolded in column 1 = significant at p<0.05)

Test		Test statistic	df	p-value	Adjusted R^2
**Body mass: adult comparisons** (Fig. 1A)<br>**Mallard-hybrid**<br>**Runner-hybrid**<br>**Runner-mallard**	One-way ANOVA + Tukey post hoc ("aov" and "TukeyHSD" functions in R)	44.68	2,48	<0.001<br><0.001<br><0.001<br><0.001	
**Body mass: growth** (Fig. 1A, 1B)	Logistic growth curves ("nls" ("logis") function in R)			All model parameters for all groups: <0.001	
**Wing area: adult comparisons** (Fig. 2A)<br>**Mallard-hybrid**<br>**Runner-hybrid**<br>Runner-mallard	One-way ANOVA + Tukey post hoc	10.82	2,38	<0.001<br><0.001<br><0.01<br>0.14	
**Wing area: growth** (Fig. 2A, 2B)	Logistic growth curves			All model parameters: <0.001	
**Wing loading: adult comparisons** (Fig. 2C)<br>Mallard-hybrid<br>**Runner-hybrid**<br>**Runner-mallard**	One-way ANOVA + Tukey post hoc	26.22	2,33	<0.001<br>0.93<br><0.001<br><0.001	
**Log wing area vs log body mass: <19 days** (all birds) (Fig. 2D)	Linear regression ("lm" function in R)	261.2	1,49	Slope (0.65), intercept: <0.001, <0.001	0.84
**Log wing area vs log body mass: ≥19 days** (Fig. 2D)	Linear regression				
**Mallard**		694.8	1,52	Slope (3.5), intercept: <0.001, <0.001	0.93
**Hybrid**		775.3	1,91	Slope (3.1), intercept: <0.001, <0.001	0.89
**Runner**		804.8	1,79	Slope (3.5), intercept: <0.001, <0.001	0.91
**Log wing area vs log body mass vs breed: ≥19 days** (Fig. 2D)	ANCOVA ("aov" or "lm"function in R, + type III tests with "Anova" function)				
**Body mass**		896.4	1	<0.001	0.91
**Breed**		2.996	2	0.05 (differences in intercepts significant)	
**Body mass : breed interaction**		2.958	2	0.05 (mallard and runner slopes differ from hybrid slope)	
No interaction (slopes identical):					
**Body mass**		2185.5	1	<0.001	0.91
**Breed**		161.0	2	<0.001 (differences in intercepts still significant)	
**Feather area: growth** (Fig. 2B)	Logistic growth curve			All model parameters: p<0.001	
**Total & primary feather area: adult comparisons** (Fig. 2E)<br>Mallard-hybrid<br>**Runner-hybrid**<br>**Runner-mallard**	One-way ANOVA + Tukey post hoc	0.727<br>7.003	2,17<br>2,17	Total feather area: 0.498<br>Primary #8 feather area: <0.01<br>0.999<br><0.01<br>0.03	
Log feather area vs log body mass: all adults (Fig. 2E)	Linear regression	0.8586	1,17	Slope (-0.09), Intercept: 0.367, <0.001	-0.0079
**Wing bones: growth** (>15 days due to delayed growth) (Fig. 2B)	Logistic growth curves			All model parameters: p<0.001	
**Wing bones: adult comparisons** (Fig. 2F)<br>**Mallard-hybrid**<br>**Runner-hybrid**<br>**Runner-mallard**	One-way ANOVA + Tukey post hoc	47.19	2,26	<0.001<br>0.01<br><0.001<br><0.001	

Test		Test statistic	df	p-value	Adjusted R^2
**Log wing bone length vs log body mass: <17 days** (Mallard only) (Fig. 2G)	Linear regression	38.06	1,12	Slope (0.24), Intercept: <0.001, <0.001	0.74
**Log wing bone length vs log body mass: 17-43 days** (Mallard only) (Fig. 2G)		142.7	1,15	Slope (1.13), Intercept: <0.001, 0.001	0.90
**Log wing bone length vs log body mass: ≥ 43 days** (all birds grouped; no visual distinctions) (Fig. 2G)		139.5	1,55	Slope (0.35), Intercept: <0.001, <0.001	0.71
**Wing muscle mass (% body mass): growth** (Fig. 2H)	Logistic growth curves			All model parameters: p<0.001	
**Wing muscle mass (% body mass): adult comparisons** (Fig. 2H)	One-way ANOVA + Tukey post hoc	21.36	2,26	<0.001	
**Mallard-hybrid**				0.01	
**Runner-hybrid**				<0.01	
**Runner-mallard**				<0.001	
**Wing muscle mass (g): growth** (Fig. 2I)	Logistic growth curves			All model parameters: p<0.001	
Wing muscle mass (g): adult comparisons (Fig. 2I)	One-way ANOVA + Tukey post hoc	2.199	2,26	0.13	
Mallard-hybrid				0.24	
Runner-hybrid				0.16	
Runner-mallard				0.99	
**Log wing muscle mass vs log body mass: <19 days** (all birds grouped) (Fig. 2J)	Linear regression	587.7	1,17	Slope (1.22), Intercept: <0.001, <0.001	0.97
**Log wing muscle mass vs log body mass: ≥19 days** (Fig. 2J)	Linear regression				
**Mallard**		381.2	1,29	Slope (2.91), Intercept: <0.001, <0.001	0.93
**Hybrid**		184.4	1,15	Slope (3.06), Intercept: <0.001, <0.001	0.92
**Runner**		271.9	1,24	Slope (2.91), Intercept: <0.001, <0.001	0.92
**Log wing muscle mass vs log body mass vs breed: ≥19 days** (Fig. 2J)	ANCOVA				
**Body mass**		851.82	1	<0.001	0.93
**Breed**		36.951	2	<0.001 (differences in intercepts significant when slopes are identical)	
Body mass : breed interaction		0.144	2	0.866 (differences in slope not significant)	
**Controlled Flapping Descent: adult comparisons** (Fig. 3A)	One-way ANOVA + Tukey post hoc	7.793	2,38	0.001	
Mallard-hybrid				0.85	
**Runner-hybrid**				<0.01	
**Runner-mallard**				0.02	
**Vertical takeoff score: adult comparison** (Fig. 3D)	One-way ANOVA + Tukey post hoc	43.65	2,42	<0.001	
**Mallard-hybrid**				<0.001	
**Runner-hybrid**				<0.001	
**Runner-mallard**				<0.001	
**Foot area: growth** (Fig. 4A, 4B)	Logistic growth curves			All model parameters: p<0.001	
**Foot area: adult comparisons** (Fig. 4A)	One-way ANOVA + Tukey post hoc	40.76	2,35	<0.001	
**Mallard-hybrid**				0.04	
**Runner-hybrid**				<0.001	
**Runner-mallard**				<0.001	
**Foot loading: adult comparisons** (Fig. 4C)	One-way ANOVA + Tukey post hoc	4.502	2,33	0.019	
**Mallard-hybrid**				0.015	
Runner-hybrid				0.26	
Runner-mallard				0.21	

Test		Test statistic	df	p-value	Adjusted R^2
**Log foot area vs log body mass: log mass < 2.95** (Fig. 4D)	Linear regression				
**Mallard**		419.9	1,24	Slope (0.66), Intercept: <0.001, <0.001	0.94
**Hybrid**		1788	1,24	Slope (0.68), Intercept: <0.001, <0.001	0.99
**Runner**		1028	1,37	Slope (0.69), Intercept: <0.001, <0.001	0.96
**Log foot area vs log body mass vs breed: log mass < 2.95** (Fig. 4D)	ANCOVA				
**Body mass**		1169.0	1	<0.001	0.97
**Breed**		8.1105	2	<0.001 (differences in intercepts significant when slopes are indentical: mallards differ from hybrids (p<0.001) but runners do not (p=0.22))	
Body mass : breed interaction		0.2541	2	0.78 (differences in slopes not significant)	
**Log foot area vs log body mass: log mass > 2.95, all breeds** (fitting separately reduces R^2) (Fig. 4D)	Linear regression	268.8	1,182	Slope (0.54), Intercept: <0.001, 0.02	0.59
**Leg length: growth** (Fig. 4E, 4F)	Logistic growth curves			All model parameters: p<0.001	
**Leg length adult comparisons** (Fig. 4E)	One-way ANOVA + Tukey post hoc	60.78	2,26	<0.001	
**Mallard-hybrid**				0.001	
**Runner-hybrid**				<0.001	
**Runner-mallard**				<0.001	
**Log leg length vs log body mass: all ages** (Fig. 4G)	Linear regression				
**Mallard**		2522	1,49	Slope (0.31), Intercept: <0.001, <0.001	0.98
**Hybrid**		341.4	1,17	Slope (0.29), Intercept: <0.001, <0.001	0.95
**Runner**		1341	1,32	Slope (0.28), Intercept: <0.001, <0.001	0.98
**Log leg length vs log body mass vs breed** (Fig. 4G)	ANCOVA				
**Body mass**		4112.141	1	<0.001	0.98
**Breed**		62.995	2	<0.001 (differences in intercepts significant when slopes are identical)	
Body mass : breed interaction		4.16	2	0.02 (differences in slopes significant though minor; model assuming same slopes has smaller p-values)	
**Leg muscle mass: growth up to 55 days** (Fig. 4H, 4I)	Logistic growth curves			All model parameters: p<0.001 or 0.01	
**Leg muscle mass: decline in absolute mass from 45-55 days (peak) to 60 days (drop)?** (Mallards only — biggest sample size) (Fig. 4H)	Welch two sample t-test	2.3628	4.0035	0.04	
**Leg muscle % body mass: juvenile comparisons** (Fig. 4J)	One-way ANOVA + Tukey post hoc	6.694	2,26	<0.01	
Mallard-hybrid				0.14 (but very small sample size for hybrids so likely a true difference, given high similarity between Runners & Hybrids)	
Runner-hybrid				1	
**Runner-mallard**				<0.01	
**Leg muscle % body mass: decline from ~30 days (peak) to 58-63 days (drop)?** (Fig. 4J)	Welch two sample t-test				
**Mallard**		6.1098	7.0691	<0.001	
**Hybrid**		4.8636	4.4797	<0.01	
**Runner**		8.5866	6.6509	<0.001	

Test		Test statistic	df	p-value	Adjusted R^2
**Leg muscle % body mass: gain after 58-63 days?** (Fig. 4J)	Welch two sample t-test				
Mallard		-0.46767	6.7823	0.6727	
**Hybrid**		3.1801	2.6643	0.03	
**Runner**		5.8946	11.791	<0.001	
**Leg muscle % body mass: adult comparisons** (Fig. 4J)	One-way ANOVA + Tukey post hoc	4.456	2,26	0.02	
Mallard-hybrid				0.14 (probably significant with larger sample size)	
Runner-hybrid				0.48	
**Runner-mallard**				0.02	
**Log leg muscle mass vs log body mass** (Fig. 4K)**:**	Linear regression				
**Mallard (<41 days)**		2083	1,30	Slope (1.10), Intercept: <0.001, <0.001	0.99
**Hybrid (<47 days)**		552.3	1,4	Slope (1.08), Intercept: <0.001, <0.001	0.99
**Runner (<51 days)**		2270	1,13	Slope (0.98), Intercept: <0.001, <0.001	0.99
**Runner (>51 days)**		30.34	1,17	Slope (0.80): <0.001	0.62
**Log leg length vs log body mass vs breed** (Mallards & Runners < 51 days; not enough small Hybrids) (Fig. 4K)	ANCOVA				
**Body mass**		2521.1	1	<0.001	0.99
Breed		8.1149	1	<0.01 (differences in intercepts significant but due to differences in slope)	
**Body mass : breed interaction**		12.406	1	<0.01 (differences in slopes significant: mallard steeper)	
**Running velocity: declines in later ontogeny?** (Fig. 5A)	Welch two sample t-test				
**Mallard**		10.31	43.552	<0.001	
**Hybrid**		6.6105	37.431	<0.001	
**Runner**		3.6966	12.602	0.001	
**Running velocity: juvenile comparisons** (10 fastest trials per group between 23-39 dph) (Fig. 5A)	One-way ANOVA + Tukey post hoc	35.23	2,27	<0.001	
**Mallard-hybrid**				<0.001	
**Runner-hybrid**				<0.01	
**Runner-mallard**				<0.001	
**Running velocity: adult comparisons** (Fig. 5A)	One-way ANOVA + Tukey post hoc	16.17	2,58	<0.001	
**Mallard-hybrid**				<0.001	
**Runner-hybrid**				<0.001	
Runner-mallard				0.61	

Table S2: Body mass

	Adult mass (g)	Variation in (adult) mass (1 SD)	Peak growth rate (g / day)	Age (days) at peak growth rate	Growth period (days)
**Runner Ducks**	1537 *Males ~ 68g heavier*	209 (14%)	41	27	86
**Hybrids**	1286 *Males ~ 14g heavier*	123 (10%)	39	24	79
**Mallards**	1068 *Males ~ 77g heavier*	99 (9%)	34	24	72

Adult masses: average of male and female averages

Variation: 1 standard deviation of adult masses (fairly even mix of males and females)

Growth rate: derivative of logistic growth curve (all model parameters statistically significant, $p<0.001$); age at peak growth rate coincides with inflection point of logistic growth curve

Growth period: day at which growth rate becomes <0.25 g/day

Table S3: Wing area & loading

	Adult wing area (cm^2, 1 wing)	Variation in (adult) wing area (1 SD)	Onset of wing growth (days)			Peak rate of wing growth (cm^2 / day)	Offset of wing growth (days)	Total growth period (days)	Adult wing loading (g/cm^2)	Variation in adult wing loading (1 SD)	Loading compared to Mallards
			Age at peak wing loading	Pin feathers emerge	Wing growth > 2 cm^2 / day						
**Runner Ducks**	501.0 *Males ~ 2% larger*	41.6 (8%)	15	~ 19, but varies by color	20	19.5 at 45 days	89	69	1.47 *Males ~1% lower*	0.15 (10%)	28% higher
**Hybrids**	547.1 *Males ~ 5% larger*	29.1 (5%)	15	~19	18	21.1 at 43 days	86	68	1.18 *Males ~4% lower*	0.098 (8%)	2% higher (not sig)
**Mallards**	465.2 *Males ~ 9% larger*	28.6 (6%)	15	~17-19	17	19.0 at 41 days	81	64	1.15 *Males ~3% lower*	0.10 (9%)	

Adult area: average of male and female averages — nearly identical to average of whole group (on graph)

Variation: 1 standard deviation of adult areas (fairly even mix of males and females)

Growth rate: derivative of logistic growth curve (all model parameters statistically significant, $p<0.001$); age at peak growth rate coincides with inflection point of logistic growth curve

Offset wing growth: 2*inflection point of logistic growth curve (feathers retract some after cessation of growth)

Table S4: Bone length

	Adult length (mm)			Variation in adult length (1 SD)		Peak rate of growth (mm / day)		Offset of growth (days)	
	Wing	Leg	Wing/Leg	Wing	Leg	Wing	Leg	Wing	Leg
**Runner Ducks**	266 (1.16x larger than Mallards)	238 (1.24x larger than Mallards)	1.12	10.2 (4%)	11.5 (5%)	7.1 at 25 days	5.9 at 9 days	77	64
**Hybrids**	242 (1.05x larger than Mallards)	210 (1.09x larger than Mallards)	1.16	6.47 (3%)	6.29 (3%)	X	X	X	59
**Mallards**	230	192	1.20	5.72 (2%)	6.86 (4%)	6.4 at 27 days	4.7 at 10 days	77	63

Forelimb length: average length of humerus + ulna + carpometacarpus; more males measured, but based on scaling relationships, an even mix of males and females would reduce lengths by <1.2% and not change overall patterns

Hindlimb length: average length of femur + tibiotarsus + tarsometatarsus; more males measured, but based on scaling relationships, an even mix of males and females would reduce lengths by <1% and not change overall patterns

Variation: 1 standard deviation of adult lengths

Growth rate: derivative of logistic growth curve (all model parameters statistically significant, $p<0.001$); age at peak growth rate coincides with inflection point of logistic growth curve

Offset of bone growth: day at which bone growth rate becomes <0.1 mm/day

Table S5: Muscle mass

	Adult muscle mass (g)		Adult muscle mass (% body mass)		Wing/Leg	Variation in adult % muscle mass (1 SD)		Peak rate of growth (g / day)		Offset of growth (days)	
	Wing	Leg	Wing	Leg		Wing	Leg	Wing	Leg	Wing	Leg
**Runner Ducks**	309	155	18.5	9.2	2.0	2.1 (12%)	0.82 (9%)	8.3 at 47 days	4.8 at 23 days	99	~50 but regained
**Hybrids**	287	118	21.4	8.8	2.4	1.5 (7%)	0.86 (10%)	8.0 at 47 days	4.0 at 24 days	99	~50 but regained
**Mallards**	310	99	24.4	7.9	3.1	1.9 (8%)	1.2 (16%)	8.3 at 50 days	4.5 at 23 days	105	~50

Muscle masses: average of all dissected birds; more males measured than females (all groups and all ages (when sexes were known)); removing females from analyses had very little effect on averages

Forelimb muscles (left and right sides): sum of pectoralis, supracoracoideus, propatagialis brevis, deltoideus major, scapulotriceps, latissimus dorsi, humerotriceps, biceps brachii, scapulohumeralis caudalis, subscapularis, coracobrachialis posterior, subcoracoideus, coracobrachialis anterior, deltoideus minor, and antebrachial muscles (extensor metacarpi radialis, extensor pollicis longus, extensor carpi ulnaris, extensor digitorum communis, anconeus, supinator, pronator sublimis, flexor carpi ulnaris, flexor digitorum sublimis, flexor digitorum profundus, pronator profundus, entepicondyloulnaris, brachialis, ulnimetacarpalis ventralis, and extensor indicis longus)

Hind limb muscles (left and right sides): sum of iliotibialis lateralis, iliotibialis cranialis, flexor cruris lateralis pars pelvica, iliofibularis, caudofemoralis pars caudalis, caudofemoralis pars pelvica, iliotrochantericus, ischiofemoralis, femorotibialis externus, femorotibialis medius, flexor cruris medialis, puboischiofemoralis lateralis, puboischiofemoralis medialis, femorotibialis internus, ischiofemoralis, gastrocnemius (externus / lateralis, intermedius, internus / medialis), fibularis longus, tibialis cranialis, and remaining shank muscles

Variation: 1 standard deviation of % muscle masses

Growth rates: derivatives of logistic growth curves (all model parameters statistically significant, $p<0.001$); ages at peak growth rates coincide with inflection points of logistic growth curves

Offset of leg muscle growth: day at which muscle mass begins to decline

Offset of wing muscle growth: day at which muscle growth rate becomes <0.1 g/day

Table S6: Foot area and loading

All data for 1 foot	Adult foot area (cm^2)	Variation in (adult) foot area (1 SD)	Onset of foot growth (days)	Peak rate of foot growth (cm^2 / day)	Offset of foot growth (days)	Total growth period (days)	Adult foot loading (g/cm^2)	Variation in adult foot loading (1 SD)	Loading compared to mallards
**Runner Ducks**	33.0 *Males ~ 8% larger*	2.5 (7%)	Immediate	0.95 at 17 days	66, but appear to continue growing slowly for a long time	66	44.4 *Males ~6% lower*	3.6 (8%)	7% higher (not significant)
**Hybrids**	27.2 *Males ~9% larger*	1.8 (6%)	Immediate	1.03 at 14 days	53	53	46.6 *Males ~5% lower*	3.1 (7%)	13% higher
**Mallards**	25.2 *Males ~2% larger*	2.4 (10%, but smaller sample size)	Immediate	0.83 at 16 days	59	59	41.4 *Males ~2% lower*	3.3 (8%)	

Foot area and foot loading: average of male and female averages

Variation: 1 standard deviation of adult areas (fairly even mix of males of females)

Growth rates: derivative of logistic growth curve (all model parameters statistically significant, $p<0.001$); age at peak growth rate coincides with inflection points of logistic growth curves

Offset of foot growth: day at which foot growth rate becomes <0.01 cm^2/day

Table S7: Allometry

	Runner Ducks, compared to Mallards	Hybrids, compared to Mallards
**Body Mass**	Peramorphosis: acceleration + hypermorphosis	Peramorphosis: acceleration + hypermorphosis
**Wing area**	No signficant change: post-displacement + hypermorphosis	Peramorphosis: acceleration + hypermorphosis
**Feather area**	No signficant change except a few distal primaries (paedomorphosis)	No signficant change
**Wing (bone) length**	Peramorphosis: acceleration	Peramorphosis
**Wing muscles**	No signficant change	No signficant change
**Foot area**	Peramorphosis: acceleration + hypermorphosis	Slight peramorphosis: acceleration + hypomorphosis
**Leg (bone) length**	Peramorphosis: acceleration	Peramorphosis: acceleration
**Leg muscles**	Peramorphosis: slight acceleration + hypermorphosis	Peramorphosis: hypermorphosis

Paedomorphosis: development of a structure is truncated, due to slower rates of development (deceleration / neoteny), earlier termination of development (hypomorphosis / progenesis), and/or later onset of development (post-displacement)

Peramorphosis: development of a structure is extended, due to faster rates of development (acceleration), delayed termination of development (hypermorphosis), and/or earlier onset of development (pre-displacement)

Movie legends

Movie S1: Controlled flapping descent (CFD) in a 6 day old Mallard

Movie S2: Controlled flapping descent (CFD) in an adult Hybrid

Movie S3: Vertical takeoff (VT) in a 14 day old Hybrid

Movie S4: Vertical takeoff (VT) in an adult Runner

Movie S5: Running in a 24 day old Hybrid

Movie S6: Steaming in a 52 day old Hybrid

References

1. Mayr, Gerald, Avian Evolution: The Fossil Record of Birds and its Paleobiological Significance (Wiley-Blackwell, 2016).
2. C. Foth, O. W. M. Rauhut, Eds., The Evolution of Feathers (Springer, 2020).
3. J. M. Starck, R. E. Ricklefs, Eds., Avian Growth and Development: Evolution Within the Altricial-precocial Spectrum (Oxford University Press, 1998).
4. A. M. Heers, K. P. Dial, From extant to extinct: locomotor ontogeny and the evolution of avian flight. Trends Ecol. Evol. **27**, 296–305 (2012).
5. K. P. Dial, A. M. Heers, T. R. Dial, "Ontogenetic and evolutionary transformations: the ecological significance of rudimentary structures" in Great Transformations in Vertebrate Evolution, K. P. Dial, N. Shubin, E. L. Brainerd, Eds. (University of Chicago Press, 2015), pp. 283–301.
6. K. P. Dial, A. M. Heers, Waxing and Waning of Wings. Trends Ecol. Evol. **36**, 457–470 (2021).
7. M. Pittman, et al., Methods of Studying Early Theropod Flight. Bull. Am. Mus. Nat. Hist. **440**, 277–294 (2020).
8. K. P. Dial, Wing-Assisted Incline Running and the Evolution of Flight. Science **299**, 402–404 (2003).
9. K. P. Dial, B. E. Jackson, P. Segre, A fundamental avian wing-stroke provides a new perspective on the evolution of flight. Nature **451**, 985–989 (2008).
10. A. M. Heers, K. P. Dial, Wings versus legs in the avian bauplan: Development and evolution of alternative locomotor strategies. Evolution **69**, 305–320 (2015).
11. B. T. Thomas, "Family Opisthocomidae (Hoatzin)" in Handbook of the Birds of the World, Vol. 3, Hoatzin to Auks, J. Del Hoyo, A. Elliott, J. Sargatal, Eds. (Lynx Edicions, 1996), pp. 24–33.
12. T. R. Dial, D. R. Carrier, Precocial hindlimbs and altricial forelimbs: partitioning ontogenetic strategies in Mallard ducks (Anas platyrhynchos). J. Exp. Biol. **215**, 3703–3710 (2012).
13. A. M. Heers, Unexpected Performance in Developing Birds. Integr. Comp. Biol. **63**, 772–784 (2023).
14. A. M. Heers, B. W. Tobalske, K. P. Dial, Ontogeny of lift and drag production in ground birds. J. Exp. Biol. **214**, 717–725 (2011).
15. H. Zollinger, "The Island of Lombok" in The Journal Of The Indian Archipelago And Eastern Asia, J. R. Logan, Ed. (Mission Press, 1851), pp. 323–344.
16. C. S. Valentine, The Indian runner duck book (1911).
17. J. A. Coutts, The Indian runner duck: its origin, history, breeding and management (1926).
18. C. Ashton, M. Ashton, The Domestic Duck (Crowood Press, 2001).
19. D. Holderread, Storey's Guide to Raising Ducks: Breeds, Care, Health, 2nd Ed. (Storey Publishing, 2011).
20. S. Sreehari, J. Yadav, Duck rearing. Indian Farming **64**, 38–41 (2014).
21. Indian Runner Duck Club I History of the Indian Runner Duck. Indian Run. Duck Club Hist. Indian Run. Duck (2023). Available at: https://runnerduck.net/history.php [Accessed 2 September 2025].
22. C. A. Schneider, W. S. Rasband, K. W. Eliceiri, NIH Image to ImageJ: 25 years of image analysis. Nat. Methods **9**, 671–675 (2012).
23. G. T. Clifton, J. A. Carr, A. A. Biewener, Comparative hindlimb myology of foot-propelled swimming birds. J. Anat. n/a-n/a. https://doi.org/10.1111/joa.12710.

24. W. R. Siegfried, Post-embryonic development of the Ruddy duck Oxyura jamaicensis and some other diving ducks. Int. Zoo Yearb. **13**, 77–87 (1973).
25. D. R. Carrier, J. Auriemma, A developmental constraint on the fledging time of birds. *Biol. J. Linn. Soc.* **47**, 61–77 (1992).
